# Visualizing hydrogen-induced reshaping and edge activation in MoS_2_ and Co-promoted MoS_2_ catalyst clusters

**DOI:** 10.1038/s41467-018-04615-9

**Published:** 2018-06-07

**Authors:** Signe S. Grønborg, Norberto Salazar, Albert Bruix, Jonathan Rodríguez-Fernández, Sean D. Thomsen, Bjørk Hammer, Jeppe V. Lauritsen

**Affiliations:** 1 Interdisciplinary Nanoscience Center (iNANO), Aarhus University, Gustav Wieds Vej 14, 8000 Aarhus, Denmark; 2 Department of Physics and Astronomy, Aarhus University, Ny Munkegade 120, 8000 Aarhus, Denmark

## Abstract

Hydrodesulfurization catalysis ensures upgrading and purification of fossil fuels to comply with increasingly strict regulations on S emissions. The future shift toward more diverse and lower-quality crude oil supplies, high in S content, requires attention to improvements of the complex sulfided CoMo catalyst based on a fundamental understanding of its working principles. In this study, we use scanning tunneling microscopy to directly visualize and quantify how reducing conditions transforms both cluster shapes and edge terminations in MoS_2_ and promoted CoMoS-type hydrodesulfurization catalysts. The reduced catalyst clusters are shown to be terminated with a fractional coverage of sulfur, representative of the catalyst in its active state. By adsorption of a proton-accepting molecular marker, we can furthermore directly evidence the presence of catalytically relevant S–H groups on the Co-promoted edge. The experimentally observed cluster structure is predicted by theory to be identical to the structure present under catalytic working conditions.

## Abstract (web summary)

Rational design of a hydrodesulfurization catalyst relies on a fundamental understanding of its working principles. Here, the authors use scanning tunneling microscopy to directly visualize and quantify hydrogen-induced reshaping and edge activation in MoS_2_ and Co-promoted MoS_2_ catalyst clusters.

## Introduction

MoS_2_ nanoclusters compose the active phase in several important heterogeneous catalytic processes, including the important industrial hydrodesulfurization (HDS) process for sulfur removal in crude oil processing^1–3^. In these catalysts, the MoS_2_ phase is present either as stacked or single MoS_2_ layers. It is well documented that due to the inertness of the MoS_2_ basal plane the catalytic activity originates from the edges of MoS_2_ layers. Optimization of the edge accessibility is therefore crucially important in catalyst preparation. The activity and selectivity is specifically enhanced in HDS catalysts synthesis by promotion of MoS_2_ by Co and Ni. The promotional effect is within the CoMoS structural model attributed to formation of new edge sites formed by preferential substitution of Co at Mo positions along the edges of single-layer MoS_2_ nanoclusters on a support^4–6^. The atomistic description of the morphology and edge structures of MoS_2_ and CoMoS-type structures has previously been investigated by scanning tunneling microscopy (STM)^7,8^ and scanning transmission electron microscopy^9,10^. The edge structures are well supported by theoretical modeling^11–13^, however, these mainly reflect the equilibrium morphology and edge structures under synthesis conditions. It is, therefore, still an open question exactly how the active clusters actually respond to the reactive HDS atmosphere at elevated hydrogen pressure and temperature. Theoretical studies predict that the edge coverage of S and H on the MoS_2_ and CoMoS nanoclusters assume variable values depending on the reaction conditions^14–17^. It is even probable that the overall nanocluster morphology of single-layer MoS_2_ and CoMoS may depend on the H_2_/H_2_S gas pressure and composition^18–21^, but unambiguous imaging of the exact cluster morphology under such conditions has not been carried out so far. Previous studies have shown how metallic catalyst clusters expose dynamic morphology in response to changes in its environmental gaseous composition^22,23^, but similar effects are not as well understood for compounds such as oxides and sulfides. Such information is crucially important for the understanding of the catalytic pathways of HDS since the elementary steps are strongly influenced by the state of the edge sites^15,24,25^. Reduced sulfur coverages are needed for strong interaction with S-bearing molecules, such as thiophene within the crude oil, whereas S–H groups formed by H_2_ dissociation on the edges drive the hydrogenation steps that precede the S extrusion process in HDS of large, sterically hindered molecules in crude oil, such as 4,6-dimethyl-dibenzothiophene (DMDBT).

In this article, we use experimental STM studies of a gold-supported model system to directly reveal the sensitivity of both the cluster shape and edge structures of MoS_2_ and promoted CoMoS nanoclusters toward reductive conditions induced by H_2_ gas dosing at elevated temperature. Applying these conditions together with precise theoretical modeling of the supported MoS_2_ and CoMoS nanocluster properties, allows us to go beyond previous atom-resolved microscopy studies to clarify how the S and H-edge coverages may change as a function of sulfo-reductive conditions. The Brønsted acid nature of the terminal S–H groups is furthermore probed in a direct way by pyridine adsorption, which acts as a molecular marker that identifies the H position on the active edges. Finally, we specifically address the influence of the gold model substrate, and discuss the relevance of our finding in relation to the general effect of a support in MoS_2_ and CoMoS catalyst clusters.

## Results

### Hydrogen-induced crystal reshaping

**Fig. 1:**
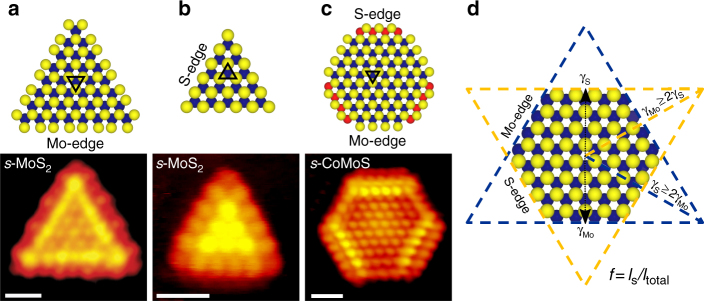
Top view ball models and atom-resolved STM images of fully sulfided nanoclusters^7,8^. **a** Triangular *s-*MoS_2_ nanocluster terminated by Mo-edges. **b** Triangular *s-*MoS_2_ nanocluster terminated by S-edges, which is representative only for edge length with less than six Mo atoms, *n* < 6. **c** Hexagonal *s-*CoMoS nanocluster terminated with unpromoted Mo-edges and Co-substituted S-edges. All scale bars are 1 nm. Color code: S: yellow, Mo: blue, Co: red. Black triangles in the basal plane are showed to emphasize the overall orientation of the lattice. **d** Two-dimensional Wulff construction representing a hypothetical hexagonal MoS_2_ nanocluster together with the definition of the shape factor *f* used in this study, where *l*_s_ is the accumulated length of the S-edges, and *l*_total_ is the total edge length

In order to gain access to the morphology and edge structures by atom-resolved STM, we synthesized single-layer MoS_2_ and Co-promoted MoS_2_ nanoclusters on an Au(111) support by methods developed previously^7,26^ (see also Methods). Atom-resolved STM images of the resulting fully sulfided MoS_2_ and CoMoS nanoclusters (termed *s*-MoS_2_ and *s*-CoMoS) are illustrated in Fig. 1a–c together with a ball model of their atomic structure^7,27^. Both types of clusters contain a bright brim in the atomic row adjacent to the outermost edge protrusions, which is a well-known one-dimensional electronic effect^27^. The shape is an important parameter of these nanoclusters as it determines the type of edges exposed in the catalyst clusters. For a single-layer MoS_2_ cluster, the shape is determined by the relative stability of the most stable low-index edges, which are usually the ($\bar 1010$) S-edges and ($10\bar 10$) Mo-edges, respectively (Fig. 1d). Unpromoted MoS_2_ nanoclusters larger than ~2 nm adopt a perfect triangular shape terminated by the Mo-edges (Fig. 1a)^27^, reflecting that this edge is significantly more stable than the S-edge under the sulfiding conditions of the synthesis^18^. It is possible to distinguish the Mo-edge and S-edge terminated clusters from atom-resolved STM images (Fig. 1a, b). The overall orientation of the triangular cluster on the substrate does not directly reflect the nature of the edge termination as the MoS_2_ clusters can grow in two different orientations due to the alternating stacking domains of the reconstructed Au substrate. Addition of Co promoters in MoS_2_ leads to hexagonally shaped CoMoS clusters terminated by both Mo- and S-edges (Fig. 1c). The modification to a truncated equilibrium shape (almost hexagonal) compared to the pure triangular MoS_2_ shape was concluded to be driven by the preferential substitution of Co atoms at the S-edges (Fig. 1c), which stabilizes the Co-substituted S-edge energy relative to the unpromoted case^7,28^. The strong promotion seen for the HDS catalyst by Co is thus in the CoMoS model attributed to the formation of the new Co S-edge sites. Our previous analysis^7^ shows that the Co-promoted S-edge can be clearly identified in STM images of the fully sulfided structures. This is due to the intense STM contrast in the brim region of the S-edge (Fig. 1c) compared with the Mo-edges, which are unpromoted and, hence, imaged identically to those of the Mo-edge in the MoS_2_ triangles (Fig. 1a).

**Fig. 2:**
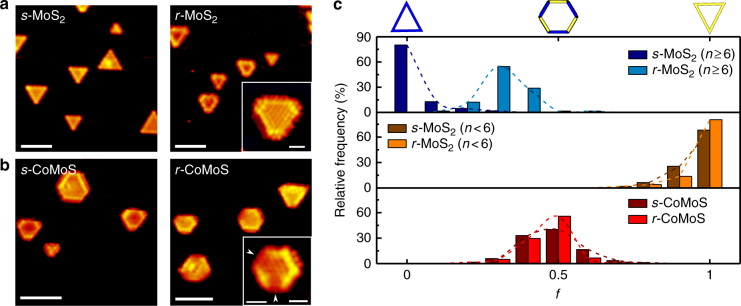
Analysis of hydrogen-induced reshaping of MoS_2_ and CoMoS. **a** The morphology of sulfided (*s*-MoS_2_) and reduced (*r*-MoS_2_) MoS_2_ nanoclusters, insert showing high-resolution image of a *r*-MoS_2_ cluster and **b** the morphology of sulfided (*s*-CoMoS) and reduced (*r*-CoMoS) CoMoS nanoclusters, insert showing high-resolution image of *r*-CoMoS cluster and white arrows point toward the quenched S-edges. All scale bars of the main images are 4 nm, the scale bar of the inserts are 1 nm. **c** Histograms of the shape factor (*f* *=* *l*_S_/*l*_total_) for sulfided and reduced MoS_2_ and CoMoS structures. Mo-edge terminated (*n* ≥ 6) and S-edge terminated (*n* < 6) MoS_2_ nanoclusters are plotted separately. The histograms were obtained from analysis of atom-resolved STM images of individual MoS_2_ and CoMoS nanoclusters (number of clusters evaluated: *s*-MoS_2_ (*n* ≥ 6): 172, *r*-MoS_2_ (*n* ≥ 6): 66, *s*-MoS_2_ (*n* < 6): 94, *r*-MoS_2_ (*n* < 6): 51, *s*-CoMoS: 85, *r*-CoMoS_2_: 61. For further statistical details, see Methods)

Figure 2a illustrates a pronounced morphological change for unpromoted MoS_2_ upon switching from a sulfiding gas to a reducing gas. The reshaping is induced by exposing the as-synthesized fully sulfided samples (*s*-MoS_2_) to H_2_ gas at a partial pressure *p*(H_2_) of 10^−4^ mbar at 400 °C for 30 min (*r*-MoS_2_). This pressure is two orders of magnitude higher than previous STM studies examining the effect of H_2_^21^, and sets a chemical potential of sulfur close to that under HDS conditions (vide infra). Indeed, the elevation in pressure to 10^−4^ mbar is clearly enough to overcome kinetic barriers and induce significant changes not observed using a lower pressure of H_2_ or pre-dissociated H^26,29^. From inspection of the large-scale STM images in Fig. 2a, we conclude that the MoS_2_ cluster shape has changed from triangular to truncated triangular and must, from symmetry, contain both the original Mo-edge and newly formed S-edges. As seen from the atom-resolved inset in Fig. 2a, the newly formed S-edge (short edges in the cluster) differs from the Mo-edges in the sense that its edge protrusions are better resolved and its brim is slightly clearer as previously reported for S-edges of non-promoted MoS_2_^21^. Hence, we are able to distinguish the two edge types allowing us to make a statistic analysis of the relative content of each edge type (S- and Mo-edge) before and after the reduction in order to quantify the degree of reshaping. We quantify the observed reshaping in terms of the cluster shape factor (*f* = *l*_S_/*l*_total_, Fig. 1d). The shape factor is a value that expresses the length of the S-edges relative to the total cluster perimeter length (sum of Mo- and S-edges) for each individual cluster and is extracted from an extensive data set consisting of edge lengths measured in atom-resolved STM images of individual clusters (see Methods). The graph of the shape factor in Fig. 2c shows that for MoS_2_ nanoclusters with a size corresponding to edges with *n* > 6 Mo atoms pr. edge (about 2 nm edge length), triangles (*f* *=* 0) strongly dominate for the fully sulfided samples (dark blue bars). The effect of reducing conditions then shifts the distribution of shapes toward truncated shapes with the peak *f* ≈ 0.3 (light blue bars) and no perfect triangles remain. A detailed comparison shows that the cluster size distribution remained unchanged after the exposure (Supplementary Figure 1). The reshaping process is therefore an isolated restructuring of each MoS_2_ nanocluster. The reversibility of the reshaping process was also investigated by exposing the sample to the original sulfiding conditions in pure H_2_S at 10^−6^ mbar. This resulfidation process resulted in the expected shift back toward lower-shape factors consistent with a higher stability of the Mo-edge under sulfiding conditions (Supplementary Figure 2). The initial almost perfect triangular morphology (*f* = 0) was, however, not regained, which suggests that kinetic effects are more predominant for the reverse reshaping process. Our experiment thus shows that the shape of single-layer MoS_2_ nanoclusters is intrinsically a dynamic property as a function of the sulfiding/reducing conditions and that the MoS_2_ cluster shape under very sulfo-reductive conditions is best represented by a truncated triangle.

The promoted catalysts consisting of CoMoS nanoclusters (*s*-CoMoS) were exposed to the same hydrogen environment at 400 °C (Fig. 2b). The resulting structure (*r*-CoMoS) contains Mo- and S-edges, which appear comparable to the *s*-CoMoS case. However, some *r*-CoMoS S-edges appear significantly different as they completely lack the bright brim along the edge (see the two marked S-edges in the high-resolution insert in Fig. 2b). The ability to distinguish S- and Mo-edges in both *s*- and *r*-CoMoS allows us once again to make a statistical analysis of the cluster shape, which is necessary to conclude whether or not an overall shape change occurs. For a large number of individual CoMoS clusters from various areas of the sample, the length of each edge was measured and the clusters’ shape factor was calculated. The statistical distribution of the shape factor, *f*, (plotted in Fig. 2c, lower panel) surprisingly suggests no statistically significant shape change between the *s-*CoMoS (dark red bars) and *r-*CoMoS (bright red bars), which both appear to reflect almost perfect hexagons (*f* ≈0 .5). The hexagonal shape of the CoMoS nanocluster is therefore concluded to be the stable equilibrium shape in both cases.

The different sensitivities of the morphology for MoS_2_ and CoMoS to the change in conditions can be rationalized in terms of the relative ratio of edge-free energies for MoS_2_ (γ_S_/γ_Mo_) (Fig. 1d). The two-dimensional Wulff construction for a single-layer MoS_2_ slab results in triangular shapes if γ_S_ ≥ 2γ_Mo_. Hence, reshaping can be induced by changes in the relative edge stability. Such changes in the free energies can take place by reduction of the terminal S coverage on the edges or adsorption of H (in the form of S–H groups), which are processes that are predicted by theory to occur under reducing conditions (e.g., in refs. ^13,18,21^). The absence of a hydrogen-induced reshaping for CoMoS, on the other hand, may be explained by the already favorable free energy of the Co-promoted S-edges, whose relative stability with respect to the unpromoted Mo-edges is apparently not further affected by the sulfo-reductive conditions. For the Co-promoted S-edge, the γ_S_/γ_Mo_ ratio is already close to unity (*f* ≈ 0.5) and no change in this shape is observed to occur leading to the conclusion that the distinct hexagonal shape of CoMoS is stable over a wide range of conditions. It is noted that MoS_2_ clusters with a size <2 nm (*n* <6) are shown separately in the statistical material in Fig. 2c (middle panel). These very small MoS_2_ nanoclusters are also triangular under sulfiding conditions, but they are already terminated in S-edges (adopting a shape factor of *f* ≈ 1) and therefore lower in S content^8^ (Fig. 1b). This shape distribution is insensitive to the hydrogen exposure as the shape factors remain identical in Fig. 2c, which is in full accordance with the fact that a further stabilization of the S-edge relative to the Mo-edge would not affect the shape of an already S-edge-terminated triangle. Small *r*-MoS_2_ nanoclusters (*n* ≤ 6) hence remain a perfectly S-edge-terminated structure (*f* ≈ 1), whereas large *r*-MoS_2_ nanoclusters (*n* ≥ 6) are terminated in a combination of Mo- and S-edge (*f* ≈ 0.3). No similar size effect is observed for neither *s-* nor *r-*CoMoS nanoclusters as the Co substitution appears to be the dominating factor of the relative edge stability of CoMoS.

**Fig. 3:**
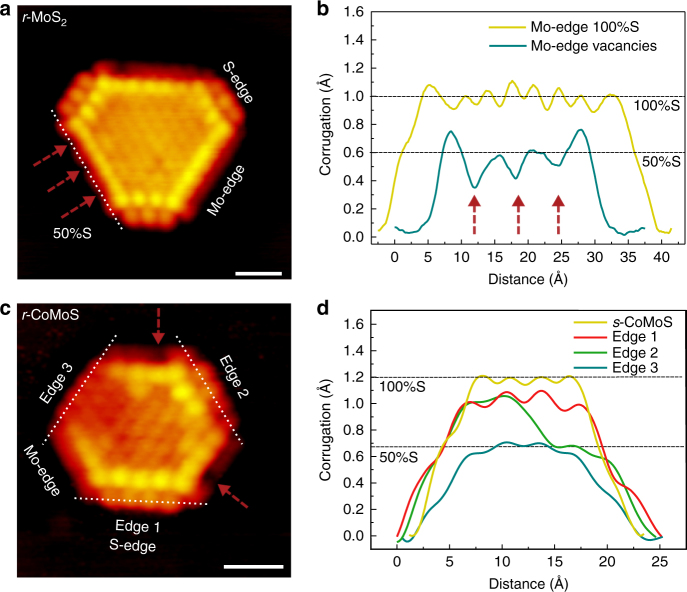
Analysis of edge structures present under sulfo-reductive conditions (*r*-MoS_2_ and *r*-CoMoS). **a** Atom-resolved STM image of an *r*-MoS_2_ nanocluster (*V*_t_ = −0.32 V, *I*_t_ = −0.42 nA). **b** Line scans along the Mo-edge as indicated on the *r*-MoS_2_ image compared with a line scan on *s*-MoS_2_. Red arrows indicate the position of S vacancies within the otherwise 50% S-covered Mo-edge. Both scale bars are 1 nm. **c** Atom-resolved STM image of an *r*-CoMoS nanocluster (*V*_t_ = −0.26 V, *I*_t_ = −0.30 nA). Red arrows again indicate sulfur vacancies in the 50% S-covered Mo-edges. **d** Line scans along the Co-promoted S-edge as indicated on the *r*-CoMoS. For reference, a line scan of the S-edge of an *s*-CoMoS cluster is also included

**Fig. 4:**
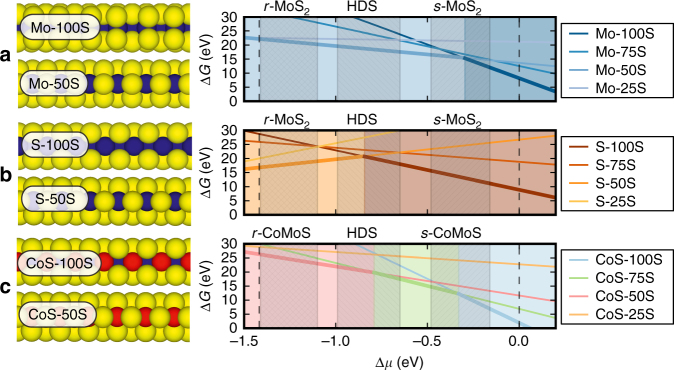
Ball models and calculated edge stabilities of Au-supported MoS_2_ and CoMoS nanoclusters. **a** Mo-edge, **b** S-edge, and **c** CoMoS S-edge terminations of MoS_2_ and CoMoS nanoclusters illustrating different S coverages. Mo: blue, S: yellow, Co: red. Phase diagrams (right panels) obtained by means of DFT calculations and ab initio thermodynamics analysis of Au-supported MoS_2_ and CoMoS nanocluster models (see Methods). The Gibbs free energy of formation (Δ*G*) of the nanoclusters has been evaluated for each edge type and coverage as a function of the chemical potential of sulfur (*μ*_s_). Hashed areas indicate regions of *μ*_s_ estimated to be representative of the indicated experimental conditions (i.e., reducing, sulfiding, or HDS)

We use atom-resolved STM to resolve the edge structures of the *r-*MoS_2_ and *r-*CoMoS nanoclusters following the hydrogen exposure in order to elucidate their sulfur coverages. We compare these observations with the possible equilibrium edge structures predicted in the DFT-calculated phase diagrams obtained by ab initio thermodynamics for MoS_2_ and CoMoS structures supported on Au(111). Figure 3a illustrates a representative truncated *r-*MoS_2_ cluster exposing both S-edges and Mo-edges. None of the edges in the *r*-MoS_2_ hexagon in Fig. 3a expose the STM contrast seen for the fully sulfided Mo-edge in *s*-MoS_2_ in Fig. 1a. Instead the shorter, newly introduced edges display clearly resolved edge protrusions, which are imaged in registry with the basal plane S atoms together with a strong enhancement of the intensity just behind the edge (the brim region) in Fig. 3a. These STM contrast signatures match the S-edge of MoS_2_, where the edge protrusions thus directly reflect the positions of the terminal sulfur atoms on the S-edge (see also Fig. 4b)^8,21^. The longer Mo-edges of the *r-*MoS_2_ cluster exhibit edge positions that are imaged as faint protrusions, in contrast to the Mo-edge of *s*-MoS_2_ in Fig. 1a. The line scan in Fig. 3b shows that the contrast level relative to the substrate along the edge atoms is reduced from 1 Å for the fully sulfided edge to ~0.6 Å and the corrugation along the edge is much weaker for this reduced edge state. This edge appearance indicates removal of half of the S from the Mo-edge resulting in a 50% S-terminated Mo-edge (Fig. 4a), and is fully consistent with STM simulations for such an edge termination^13,18,21,30^. In addition, we can locate a few atomic positions on the Mo-edge imaged with an even darker contrast (marked with red arrows in Fig. 3a). Their location is also clearly visible in the line scan as a further ~0.2 Å reduction in the height profile, pointing to formation of isolated sulfur vacancies corresponding to reduction below the 50% S coverage.

Calculated phase diagrams for the Mo- and S-edges for Au-supported clusters obtained by ab initio thermodynamics analysis are shown in Fig. 4. These were constructed by evaluating the Gibbs free energy of formation (Δ*G*) as a function of the chemical potential of sulfur (*μ*_s_) for nanocluster models exposing the three different edge types considered and varying S coverage (see Methods). As a compromise between computational cost and experimental compatibility, we have chosen MoS_2_ triangles with a 6 Mo atom side length (*n* = 6) to evaluate the edge stabilities (see Methods). To facilitate comparison with experiment, we superimpose stability windows representative of the experimental sulfiding conditions (*s*-MoS_2_), reducing conditions (*r*-MoS_2_), and HDS conditions, noting that the span in *μ*_s_ reflects the uncertainties in the determination of the chemical potentials from theory and the experimental conditions. An estimate of the sulfur chemical potential (*μ*_s_) achieved under the reducing conditions (*r*-MoS_2_) (see Methods) shows that the reactive atmosphere is in fact more sulfo-reductive (lower *μ*_s_) than that typical under HDS conditions, which implies that the reduced edge structures become accessible under the conditions of the experiment despite the lower pressure^31^. The Mo-edge with the 100% S coverage is stable within the range of *μ*_s_ estimated to correspond to the experimental sulfiding conditions leading to *s*-MoS_2_ (Fig. 4a). In turn, the range of *μ*_s_ defined by reducing conditions (*r*-MoS_2_) coincides with the region of the phase diagram, where the 50% S coverage on the Mo-edge becomes thermodynamically stable. This transition is in full accordance with our STM observations and agrees with previous DFT-derived phase diagrams for Au-supported stripes of MoS_2_^21^. We also note that in some STM images of *r*-MoS_2_, S vacancies could be identified on edges which otherwise bear resemblance to defective edges with 50 and 100% S coverage (Supplementary Figure 3). We attribute such observations to varying degrees of S coverage above and below 50% S on the Mo-edge, reflecting that kinetic effects are still significantly present during the transition from the fully sulfided Mo-edges when exposed to *r*-MoS_2_ conditions. For MoS_2_, we hence conclude that the Mo-edge of MoS_2_ generally is reduced from 100% S coverage (*s*-MoS_2_) to 50% S for the *r*-MoS_2_ case. For unpromoted S-edges, the calculated phase diagram (Fig. 4b) suggests that the 100% S coverage is stable for *s*-MoS_2_ conditions and for a wide range of lower *μ*_s_ values, whereas for conditions leading to *r*-MoS_2_, the lower 50% S coverage becomes accessible on the S-edge. However, we observe no difference between the S-edges under *s*-MoS_2_ (exposed in imperfect non-triangular *s*-MoS_2_ structures), and the S-edge exposed in the hexagonal *r*-MoS_2_, which suggests that such a transition does not take place in our experiment.

Since the same sulfiding and reducing experimental conditions apply for MoS_2_ and CoMoS, same *μ*_s_ ranges in the calculated phase diagrams are examined when considering edge coverages of CoMoS edges. As the Mo-edge of CoMoS is identical to the Mo-edge of MoS_2_ that same conclusion about Mo-edge S coverage is made for CoMoS (Fig. 4a). For the S-edge in CoMoS, it is noted that the phase diagram (Fig. 4c) predicts that two edge structures with 100% and 75% S are possible within the *s*-CoMoS window, which is in contrast to previously proposed models of this edge^7^. This revision is due to explicitly accounting for the interaction between finite CoMoS nanocluster models (instead of extended stripe models^7^) and the Au(111) substrate in our DFT calculations. The Au substrate results in a stabilizing effect for the high-edge sulfur coverage for both promoted and unpromoted S-edges. The periodic S-edge contrast of *s*-CoMoS in STM further suggests 100% S coverage rather than 75% S (Fig. 1c). The *s*-CoMoS structure is therefore seen to contain Co in an unusual trigonal prismatic coordination to six S atoms (hereof four edge sulfurs, see Fig. 4c). Upon applying the sulfo-reductive conditions (*r*-CoMoS region in the phase diagram, Fig. 4c), the S coverages of the equilibrium structure decrease to 75% and finally to 50% S coverage. In agreement, we experimentally observe significant changes to the edge structures indicative of sulfur reduction on the S-edge of *r-*CoMoS (Fig. 3c) relative to *s*-CoMoS (Fig. 1c). Most apparent is the loss of the strongly intense brim observed in STM images, which now appears to be quenched to a level lower than the basal plane on a large fraction of the S-edges (Edge 3 in Fig. 3c). Resulfidation in H_2_S restores the original appearance linked with the 100% S coverage in *s*-CoMoS (Supplementary Figure 4). This confirms that these quenched S-edge on *r*-CoMoS reflects the 50% S coverage predicted by DFT and shows that S adsorption/desorption (which is a key step in the HDS cycle) is facile between the 100% and 50% S coverage and fully reversible on the CoMoS S-edge under the experimental conditions.

### Probing S–H groups on CoMoS edges by pyridine titration

The structure of the *r*-CoMoS S-edges turns out to be more complex, as we also evidence the formation of S–H-edge groups, expected to be present on the catalyst in its working state. First, it is important to note that the S dimers of 100% S-covered S-edge and the monomers of a 50% S-covered S-edge are both positioned in the bridging position (ball model in Fig. 4c), so the lateral sulfur location would not change between the two coverages in STM. In the following discussion, we subdivide the *r*-CoMoS S-edges into two categories: quenched and non-quenched edges. The line scan in Fig. 3d compares the corrugation along a fully sulfided *s*-CoMoS S-edge, with the non-quenched (Edge 1 in Fig. 3c), partly quenched (Edge 2), and fully quenched edge (Edge 3) counterparts within the *r*-CoMoS cluster in Fig. 3c. Here, we note that S-edge protrusions of all Co S-edges of *r*-CoMoS clusters are imaged lower than that of *s*-CoMoS (line scan, Fig. 3d).

The distinct contrast lowering of the brim sites of the *r*-CoMoS upon reduction (brim quenching) is easily observable in large-scale STM. However, only some edges appear in the quenched state after the H_2_ reduction (Fig. 3c). As the 50% S Co S-edge is predicted to be the only stable edge configuration around the experimental reducing conditions (Fig. 4c), one would expect that all edges eventually adapt this configuration. Surprisingly, increasing the reduction time to overcome kinetic barriers did not lead to a more frequent appearance of the quenched edges (as shown in the statistical plot in Supplementary Figure 5). Instead, it is observed that increasing the reduction temperature from 300 to 400 °C only results in a faster transition into an *r*-CoMoS equilibrium state, which always includes a mixed population of quenched edge sites and sites that retain a bright brim and more intense edge protrusions.

**Fig. 5:**
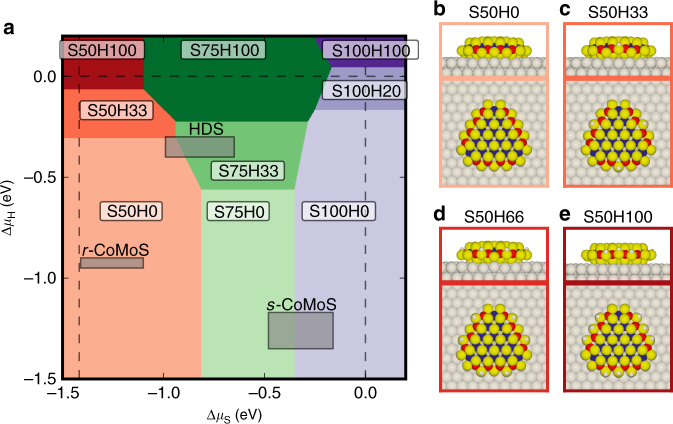
Edge structures of CoMoS. **a** Extended phase diagram for the CoMoS S-edge, which includes the chemical potential of hydrogen and sulfur to allow for S–H group formation. The markers indicate the estimated chemical potentials at conditions for *s*-CoMoS, *r*-CoMoS, and HDS, respectively. **b**–**e** Ball models of selected Au-supported CoMoS nanocluster structures, illustrating the effect of varying H coverages (0–100%) on the 50 % S S-edge. Ball models for the complete set of structures used to construct the phase diagram in **a** is shown in Supplementary Figure 7.

Interestingly, the quenched state is accompanied by a gradual lowering of the near-edge region of the nanocluster when approaching the cluster edge (e.g., Edge 3 in Fig. 3c). This is consistent with the DFT-calculated edge structures for the 50% S 0% H-edge in Fig. 5b, where the edge monomers are shifted downward and bind to the underlying support. In contrast, the terminal S atoms on the original 100% S-edge are not interacting with the substrate (Supplementary Figure 6). The quenched state is therefore attributed to the 50% Co S-edge with no H. Increasing the hydrogen coverage gradually in the calculated structures (Fig. 5c, d, e) show that any adsorption of H on the 50% S-edge lifts the edge S monomers back up from the substrate. Since no lateral shift is induced on the sulfur position along the edge due to H, there is a strong resemblance between a 100% S-edge and the 50% S-edge with H adsorbed on the terminal S monomers in STM images. Line scans (Fig. 3d) reveal that the edge sulfur atoms on the non-quenched S-edges (with S–H) of *r*-CoMoS appear ~0.2 Å lower than the S-edges of *s*-CoMoS, consistent with the geometry difference between S dimers and S monomers. This is in contrast to the significantly lowered height of the S monomers on the 50% S 0% H-quenched edges in the same figure. From these observations, we conclude that all edges in the equilibrium *r*-CoMoS are in fact reduced to a 50% S coverage. The difference in edge appearance (quenched and not quenched) arises from the absence or presence of edge-adsorbed hydrogen building up in parallel with the reduction of the S coverage, during the experiment. In addition, simulated STM images (Supplementary Figure 8) support qualitatively the loss/restoring of the brim in the case of 50% S Co S-edge without/with H, respectively.

**Fig. 6:**
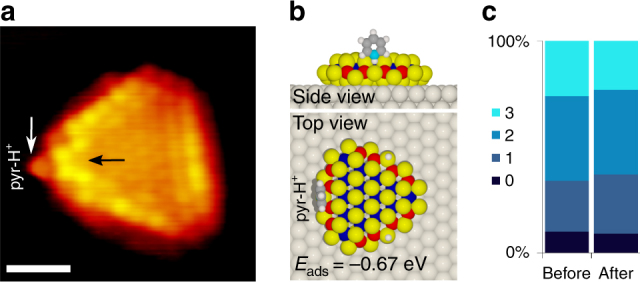
Pyridine adsorption on *r-*CoMoS. **a** Atom-resolved STM image of an *r*-CoMoS nanocluster (*V*_t_ = −0.71 V, *I*_t_ = −0.60 nA) after dosing pyridine at 300 K. Scale bar is 1 nm. **b** Calculated ball model for the most stable adsorption configuration of pyr-H^+^ on an *r*-CoMoS S-edge. The formation of pyr-H^+^ is crucial for adsorption as no stable non-protonated pyridine adsorption modes were found (see Supplementary Figure 9). Color code as previous figures, N: cyan. **c** Average distribution of quenched edges per cluster based on a cluster count for *r*-CoMoS before and after pyridine dosage (0 = no quenched edges, 3 = all edges quenched)

To detect the edge H in *r-*CoMoS in a direct way, we use pyridine (C_5_H_5_N) as a probe molecule allowing us to mark the S–H positions on the edges in atom-resolved STM images of CoMoS. Pyridine is a well-known N-containing proton acceptor, widely used in IR-spectroscopy as a probe for Brønsted acidity and used previously to investigate S–H groups on sulfides^32^. Pyridine may form strongly bound pyridinium (*pyr-H*^*+*^) upon protonation by the S–H groups on alumina-supported CoMoS catalysts, which adsorbs more strongly than un-protonated pyridine^33^. Hence, sites containing edge hydrogen species will enable the adsorption and formation of *pyr-H*^*+*^^34^, whereas interaction with the bare 50% S-edge in *r*-CoMoS is much weaker^25^. In the experiment, we prepared *s*-CoMoS and *r*-CoMoS samples and exposed these to pyridine vapor at 10^−6^ mbar at room temperature, followed by atomic resolution STM imaging at the same temperature. As expected, pyridine has no affinity for the 100% S-covered *s*-CoMoS S-edges, nor for the Mo-edge. In contrast, the atom-resolved STM image of an *r*-CoMoS nanocluster after exposure to pyridine reveals that some of the S-edges have facilitated the adsorption of the pyridine. An adsorbed pyridine molecule is identified in the STM image in Fig. 6a by the simultaneous appearance of a distinct small protrusion outside the S-edge (indicated with a white arrow) and an additional structure slightly shifted outward from the brim position (black arrow). We attribute the feature adsorbed on the edge to the facile adsorption of pyridine on S–H groups on the Co S-edge, resulting in the formation of bound *pyr-H*^*+*^. This is consistent with DFT studies showing that the formation of *pyr-H*^*+*^ on the hydrogenated 50% S Co S-edge happens with zero activation energy and is thermodynamically favorable^25^. Our DFT calculations further show that the pyridine adsorption of the Co S-edge of our Au-supported system is only favorable in the case of the formation of *pyr-H*^*+*^ ion (Fig. 6b), whereas pyridine adsorption alone on both protonated and non-protonated Co S-edges is significantly less favorable (Supplementary Figure 9). The outermost edge featured in the STM images may reflect a complex convolution of electronic and geometric structure of the adsorbed molecule, but the symmetry and size agrees well with the predicted *pyr-H*^*+*^ adsorbed across the CoMoS S-edge shown in the calculated configuration of Fig. 6b. The STM image shows that the two other S-edges remain in the quenched state, further indicating that the 50% S 0% H-covered edge is unreactive toward pyridine. In fact, we can statistically correlate the adsorption of pyridine in our STM data uniquely to the non-quenched edge part (50% S with H) of the *r*-CoMoS clusters. Figure 6c illustrates that the statistical distribution of quenched edge sites in *r*-CoMoS before and after is unaffected by the pyridine dosage, confirming that the S–H groups binding pyridine were in fact originally located on the non-quenched sites within *r*-CoMoS. The state of *r*-CoMoS in our experiment therefore has 50% S coverage on all edges, but with additional H on some of the Co-promoted S-edges.

The extended phase diagram shown in Fig. 5a includes both S and H adsorption configurations of the Au-supported CoMoS nanoclusters based on their free energies as a function of Δ*μ*_s_ and Δ*μ*_H_. The equilibrium state of the CoMoS S-edge at the high H_2_ pressures relevant to HDS conditions indeed contains fractional coverages of H (up to 33%). The lower H_2_ pressure used in the experiment to form *r*-CoMoS again predicts a 50% S coverage on the S-edge, but the calculated equilibrium state (S50H0) does not reflect H adsorbed in an intrinsically stable configuration. The S–H groups detected experimentally in *r*-CoMoS are identical to those present under HDS conditions; however, under our experimental conditions, they are a result of kinetic effects preventing the recombination of adsorbed H into gas phase H_2_. In agreement, H_2_ recombination from S–H groups on the Co S-edges has an activation barrier of 1.1 eV^35,36^, which strongly supports the presence of kinetically trapped hydrogen on the Co S-edge. We anticipate that the H becomes kinetically trapped on the edge during the 100–50% reduction of the S coverage in the transition from *s*-CoMoS to *r*-CoMoS, which necessarily involves H_2_ dissociation.

### Role of Au substrate on the stability of edge structures

Comparing the theoretically predicted stabilities of the gold-supported and -unsupported CoMoS clusters (Supplementary Figure 6) show that the 50% S coverage on the CoMoS S-edge is preferred at the chemical potential associated with the formation of *r*-CoMoS for both situations. The transition point to a higher S coverages depends only slightly on the presence of the Au and is within the HDS window for both free standing and Au-supported CoMoS. The findings are therefore also relevant for CoMoS on other weakly interacting substrates. The Mo-edge, which is expressed in both the unpromoted MoS_2_ and within the CoMoS clusters, is terminated by 50% S coverage under reducing condition in our experiment and HDS conditions regardless of the support^11,28,37^. For Au-supported CoMoS, DFT calculations show no strong bonding effect for the S atoms on either the 50% S or 100% S Mo-edge^38,39^. In contrast, the terminal S atoms on the 50% S-covered CoMoS S-edge are clearly bonded toward the substrate when no H is present (Fig. 5b–e), which is imaged in STM as a quenched edge region (Fig. 3c). The same bonding affinity to Au is found theoretically for the edge S of the unpromoted S-edge for a 50% S coverage^38^, but we never observed quenching or periodic lowering of the edge S, reflecting that a possible lower S coverage on the unpromoted S-edge was not accessible in our experiment. The gold substrate interaction will likely influence the affinity of molecular adsorption of S-bearing molecules on the CoMoS S-edge in a way that is not necessarily of the same nature as for the usual substrates used for the HDS catalyst. However, understanding such substrate effects may explain the reported differences in HDS activity and selectivity for CoMoS clusters on various supports, and the effect should be accounted for in further interplay between theory and experiment for CoMoS on the relevant oxide surfaces.

## Discussion

**Fig. 7:**
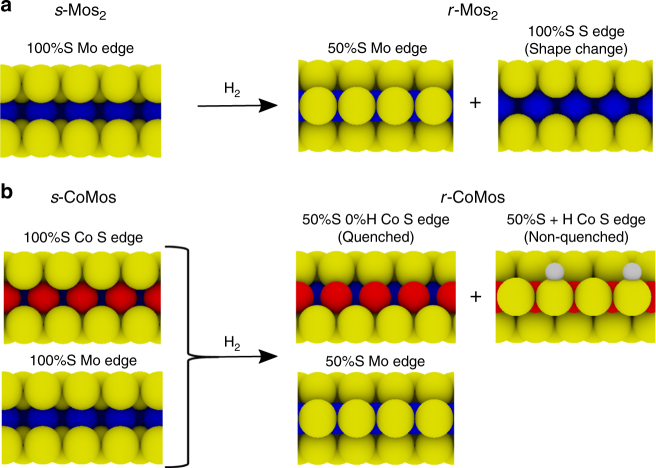
Side-view ball model providing an overview of edge structures. **a** Non-promoted *s*-MoS_2_ is purely 100% S Mo-edge terminated. After H_2_ reduction (*r*-MoS_2_) expose both Mo-edge (50% S) and S-edge (100% S). **b** *s*-CoMoS exposes both edge types, namely 100% S Mo-edge and 100% S Co S-edge, the latter being unique to CoMoS. After H_2_ reduction (*r*-CoMoS), the S content of both the Mo-edge and the Co S-edge is reduced to 50%. Some of the new, reduced 50% Co S-edges contains S–H groups (non-quenched), whereas others do not (quenched)

We report how unpromoted MoS_2_ and promoted CoMoS clusters undergo changes in cluster shape and edge coverage (S and H coverages) upon exposure to very sulfo-reductive conditions representative of those in HDS. The cluster structure under the experimental reducing conditions is significantly different from the sulfided state, and is predicted by DFT to reflect the theoretically stable phase under HDS conditions at elevated pressures. Based on our atom-resolved STM analysis and theoretical modeling, we summarize the observed edge structures in Fig. 7. The as-synthesized *s*-MoS_2_ is terminated purely by fully sulfided Mo-edges (100% S coverage) but undergoes a shape transition upon post-synthesis reduction from triangular to truncated triangular shape (*f*~0.3), which introduces of a new edge type in the cluster: the S-edge. The remaining Mo-edges generally reduce their sulfur content to 50% S coverage. The as-synthesized Co-promoted clusters, *s*-CoMoS, are initially hexagonally shaped (*f*~0.5) and terminated in both Mo-edges and Co S-edges, which are both fully sulfided (100% S covered). CoMoS undergoes no shape change upon reduction but reduces S coverage of both the Mo and the Co S-edge. Here, the latter also adapts a number of partially hydrogenated edge structures, where the edge S–H groups are experimentally detected using pyridine as a probe molecule.

As the shape analysis of the reduced clusters reveals that a truncated triangular shape (*f* ~0.3–0.5) describes both *r*-MoS_2_ and *r*-CoMoS clusters, the intrinsic distribution of Mo-edge and S-edge sites must be similar for the two reduced systems. Under reducing conditions, we can therefore conclude that the strong promotional effect of Co on HDS activity must be explained by the Co S-edge, which is unique to CoMoS.

Re-sulfidation of the reduced clusters fully restores the 100% sulfided edge structure while it only partially restores the cluster shape in the case of unpromoted MoS_2_ clusters. The adsorption and desorption of S on the cluster edges structure that best defines the HDS working state are consequently very facile even at the relative low pressures applied in the experiment. Additional adsorption of H atoms on the reduced edges in the form of S–H groups were exclusively observed for the Co-promoted S-edges, which presumably functions a key factor of the Co promotional effect on HDS activity. The calculated equilibrium structure of the Co S-edge under the range of chemical potentials corresponding to HDS working conditions (marked by a box on the phase diagram in Fig. 5a) overlap several regions with stable S coverages at or above 50% S, with and without a fractional H coverage (33% H). Such a situation is favorable catalytically as it reflects that S and H adsorbates on the edges are neither too weakly nor too strongly bound. As the Co S-edges of *r*-CoMoS contain a similar mixture of edges with and without H adsorbates (and all at 50% S coverage), the experimentally studied *r*-CoMoS structures (Fig. 3c) are concluded to represent precisely the active state of all the edges terminating the CoMoS catalyst clusters. Higher hydrogen pressures than those of the experimental conditions in our study are needed to adsorb H in a thermodynamically stable configuration, but H can be kinetically trapped on the edge following reduction in H_2_. In future studies, changes of the CoMoS morphology under H_2_ exposure monitored by spectroscopy techniques such as ambient pressure X-ray photoemission spectroscopy could provide further insight regarding the morphology change dynamics and stable configurations. We conclude that this reduced CoMoS/Au(111) model system accurately describes the active edge sites in the HDS catalyst and that this experimental procedure thus allows further studies addressing the key catalytic mechanism of adsorption and reactions of refractory S and N-bearing molecules in crude oil.

## Methods

### Scanning tunneling microcopy

The experiment is based on the synthesis method of well-defined nanocrystals of MoS_2_ and Co-promoted MoS_2_ (CoMoS) described in detail elsewhere (MoS_2_^26,40^ and CoMoS ^7,41^). The synthesis was carried out on a clean Au(111) single crystal in a standard ultra-high vacuum (UHV) installed with an Aarhus STM. Mo and Co evaporation was done by physical vapor deposition using an e-beam evaporator. H_2_S gas (99.8%) was used for sulfidation. H_2_ gas (99.999%) was used for reduction experiments. The gas composition and purity was monitored using a quadrupole mass spectrometer. The sample temperature was measured with a K-type thermocouple in contact with the backside of the Au crystal. All STM data were recorded with the sample at room temperature and under UHV conditions better than 5 × 10^−10^ mbar. The sulfiding conditions for the synthesis of *s*-MoS_2_ and *s*-CoMoS were achieved by annealing to 400 °C in an H_2_S atmosphere corresponding to ~10^−6^ mbar for 15 min. The hydrogen pressure (residual gas) was estimated to be ~10^−9^ mbar. The sample was cooled to 450 K before H_2_S was pumped away. The reducing conditions for the synthesis of *r*-MoS_2_ and *r*-CoMoS were achieved by back-filling the UHV chamber with H_2_ to a chamber pressure in excess of 10^−4^ mbar and the sample was annealed to 400 °C in the H_2_ atmosphere. The ionization gauge for pressure measurement was switched off during the hydrogen exposure and (calibrated) pressure measurement was done with a cold cathode gauge mounted in the main chamber. The H_2_S pressure in the residual gas was estimated to be 10^−8^ mbar during reducing conditions. The annealing time for the standard experiment was 30 min. The sample was cooled to 450 K before H_2_ was pumped away. For the extended experiments of varying anneal time and temperature (data in Supplementary Figure 5), a fresh *r*-CoMoS synthesis was used for every set of conditions. The resulfidation was done by exposing the sample to 10^−6^ mbar H_2_S anneal for 15 min at 400 °C, as for the initial sulfidation step. For the purpose of collecting the statistics presented in Fig. 2c, we assigned edge type (S-edge or Mo-edge) and measured the length of each edge in the individual clusters. The total length of all S-edges and Mo-edges was measured for each cluster and the shape factor, *f*, was then calculated as:

1 $$f = \frac{{{\mathrm{Total}}\,{\mathrm{length}}\,{\mathrm{of}}\,{\mathrm{all}}\,{\mathrm{S \hbox{-} edges}}\,{\mathrm{within}}\,{\mathrm{the}}\,{\mathrm{cluster}}}}{{{\mathrm{Total}}\,{\mathrm{length}}\,{\mathrm{of}}\,{\mathrm{all}}\,{\mathrm{edges}}\,\left( {{\mathrm{S \hbox{-} edge}} + {\mathrm{Mo \hbox{-} Edge}}} \right)\,{\mathrm{within}}\,{\mathrm{the}}\,{\mathrm{cluster}}}}$$

Hence, we calculate the shape factor, *f*, for each individual cluster. The number of clusters evaluated in each of the six cases of Fig. 2c is described in Table 1.

**Table 1:** Scheme of the statistical details on the data of shape factor, *f*, calculations

Cluster type	Number of clusters evaluated	Average *f*	Standard deviation
*s*-MoS_2_ (*n* > 6)	172	0.020	0.052
*r*-MoS_2_ (*n* > 6)	66	0.271	0.075
Small *s*-MoS_2_ (*n* ≤ 6)	94	0.945	0.083
Small *r*-MoS_2_ (*n* ≤ 6)	51	0.961	0.078
*s*-CoMoS	85	0.430	0.095
*r*-CoMoS	61	0.412	0.071

For pyridine (C_5_H_5_N) dosage, the liquid (pyridine anhydrous, 99.8% purity, Sigma-Aldrich) was kept in a glass container and admitted from its vapor phase into the UHV chamber through a leak valve connected to a stainless steel tube directed to the sample surface. Prior to the exposure, the pyridine was purified by several freeze-pump-thaw cycles to remove impurities. The pyridine exposure was carried out on a sample at room temperature at a background pressure of 1.0 × 10^−7^ mbar for 5 min.

### DFT calculations

The DFT calculations of the Au-supported MoS_2_ and CoMoS nanocluster models were carried out using the optB88-vdw exchange-correlation functional^42^, the projector-augmented wave method of Blöchl^43^, and a real-space grid with spacing for the expansion of the wave functions as implemented in the GPAW code^44^ and supported by the atomic simulation environment^45^. The geometries for the different models used were optimized until the forces on each relaxed atom were lower than 0.025 eV Å^−1^ and the electronic structure at each geometry optimization step was self-consistently converged with energy, density, and eigenvalue thresholds of 5E−4, 1E−4, and 5E−8 eV, respectively. A grid spacing of 0.175 Å was used and the reciprocal space was sampled using only the gamma point. As a compromise between computational cost and compatibility with experiment, we have chosen MoS_2_ triangles with a six Mo atom side length *n* = 6 terminated either by S-edge of Mo-edges^46^. The calculated model for the CoMoS cluster is shown in Supplementary Figure 6. All the atoms of the nanocluster and the upper layer of the two-layer slab used to represent the Au(111) substrate were allowed to relax during geometry optimization. The Au slab was constructed using a lattice parameter 2.1% smaller than the optimized value in order to reproduce the compression of the Au atoms in the herringbone-reconstructed Au(111). This compression was found to lead to better agreement with experiments for Au-supported MoS_2_ nanoclusters^38^.

The Au supercell size used for supporting the CoMoS nanocluster corresponds to a matrix notation of $${\left( {\begin{array}{*{20}{c}} { - 5} & {10} \\ 6 & 4 \end{array}} \right)}$$ with 80 Au atoms per surface layers, which ensures that the distance between CoMoS nanoclusters in neighboring cells is at >7 A in all directions. For MoS_2_ nanoclusters, a smaller Au supercell with a matrix notation $${\left( {\begin{array}{*{20}{c}} { - 4} & 8 \\ 6 & 4 \end{array}} \right)}$$ resulting 64 Au atoms per layer was used, ensuring >4 A distances between clusters in neighboring cells. While these distances may not completely eliminate interaction with nanoclusters in neighboring cells, such weak interaction does not affect the resulting phase diagrams of the supported clusters.

Phase diagrams and ab initio thermodynamics: The phase diagrams for the supported nanoclusters were constructed following previous work^28,47^ by calculating the Gibbs free energy of formation (Δ*G*) as a function of the chemical potential of sulfur. For supported MoS_2_ nanoclusters:

2 $$\Delta {G}\left( {\Delta n,\mu _{\rm{S}}} \right) = E_{{\rm{MoS}}_2}^{{\rm{DFT}}} - n_{{\rm{Mo}}}E_{{\rm{MoS}}_2}^{{\rm{ref}}} - \Delta n\Delta \mu _{\rm{S}} - E_{{\rm{Au}}}^{{\rm{DFT}}} - n_{\rm{H}}\Delta \mu _{\rm{H}}$$

where

3 $$\Delta n = \,2n_{{\rm{Mo}}} - n_{\rm{S}}$$

4 $$\Delta \mu _{\rm{S}} = \,\mu _{\rm{S}} - E_{{\rm{S - bulk}}}^{{\rm{DFT}}}$$

5 $$\Delta \mu _{\rm{H}} = \,\frac{1}{2}\left( {\mu _{{\rm{H}}_2} - E_{{\rm{H}}_2}^{{\rm{DFT}}}} \right)$$

Here, $E_{{\rm{MoS}}_2}^{{\rm{DFT}}}$ is the DFT-calculated energy of the supported MoS_2_ nanocluster, $E_{{\rm{MoS}}_2}^{{\rm{ref}}}$ is the DFT-calculated energy of the reference bulk MoS_2_, and $E_{{\rm{Au}}}^{{\rm{DFT}}}$ is the DFT-calculated energy of the bare Au(111) slab. *n*_Mo_ and *n*_s_ correspond to the number of Mo and S atoms, respectively, of the MoS_2_. Δ*n* therefore represents the excess or deficiency of S atoms of the nanoclusters with respect to the stoichiometric 2:1 S:Mo ratio, which will change for different edge coverages. *n*_H_ is the number of H atoms adsorbed on the nanocluster. *μ*_s_ and *μ*_H_ depend on the gas phase conditions, and can be approximated from the temperature and partial pressures of H_2_ and H_2_S using ideal-gas laws ($\mu_{\rm{S}} = \mu _{{{{\rm{H}}_2{\mathrm S}}}} - \mu_{{{{\rm{H}}_2}}}$). $E_{{{{\rm{H}}_2}}}^{{\rm{DFT}}}$ is the ZPE-corrected DFT energy of a gas phase H_2_ molecule.

For the supported CoMoS nanoclusters:

6 $$\Delta {G}\left( {\Delta n,\mu _{\rm{S}}} \right) = E_{{{{\rm{Co}}-{\rm{Mo}}-{\rm{S}}}}}^{{\rm{DFT}}} - n_{{\rm{Mo}}}E_{{\rm{MoS}}_2}^{{\rm{ref}}} - \frac{{n_{{\rm{Co}}}}}{9}E_{{{{\rm{Co}}_9{\rm{S}}_8}}}^{{\rm{ref}}} - \Delta n\prime \mu _{\rm{S}} - E_{{\rm{Au}}}^{{\rm{DFT}}} - n_{\rm{H}}\Delta \mu _{\rm{H}}$$

Where:

7 $$\Delta n\prime = \,2n_{{\rm{Mo}}} + \frac{8}{9}n_{{\rm{Co}}} - n_{\rm{S}}$$

Here, $E_{{{{\rm{Co}}-{\rm{Mo}}-{\rm{S}}}}}^{{\rm{DFT}}}$ is the DFT-calculated energy of the supported CoMoS nanocluster and $E_{{{{\rm{Co}}_9{\rm{S}}_8}}}^{{\rm{ref}}}$ is the DFT-calculated energy of bulk Co_9_S_8_, which is the stable Co sulfide phase under the range of chemical potentials considered.

The chemical potentials ($\mu _{\rm{S}}$ and $\mu _{\rm{H}}$) are defined by the conditions of the gas in equilibrium with the nanoclusters and can be evaluated in terms of the temperature partial pressures of H_2_ and H_2_S^31^. The experimental synthesis, reductive, and HDS conditions thus result in the *μ*_s_ and *μ*_H_ values indicated in Table 2. Ranges of *μ*_s_ and *μ*_H_ are given instead of discrete values to consider variations due to the uncertainties in relative pressures and to the approximations used in the calculations.

**Table 2:** Conditions used for DFT calculations

Conditions	*T* (K)	*p*(H_2_S) (Pa)	*p* (H_2_) (Pa)	*μHH[eV]*	Δ*μ*_s_ (eV)
HDS	573/650	10^4^/0.5 × 10^6^	10^6^	−0.35/−0.30	−0.94/−0.65
*s*-MoS_2_/*s*-CoMoS	673	10^−4^	10^−8^/10^−6 a^	−1.30/−1.17	−0.43/−0.16
*r*-MoS_2_/*r*-CoMoS	673	10^−7^/10^−5 a^	10^−2^	−0.90	−1.36/−1.10

Scheme summarizing the conditions corresponding to HDS, *s*-MoS_2_/*s*-CoMoS, and *r*-MoS_2_/*r*-CoMoS

^a^Residual gas measurement, not measured at sample

Simulated STM images are shown in Supplementary Figure 8 for a CoMoS nanocluster with a 50% CoS-edge without and with adsorbed hydrogen. Similar to previous work^13^, these images were generated using the Tersoff–Hamann approximation with *I*_t_ = 1.7 mA and *V*_t_ = −0.8 V. The simulated STM images qualitatively capture different electronic structure features observed experimentally. In particular, the lack of a bright brim along the 50% CoS-edge without H is perfectly reproduced by the simulated STM images. For the hydrogenated states, the simulations also reproduce the slightly lighter brim and larger protrusions along the edge resulting from the presence of S-H groups.

The pyridine adsorption calculations (Supplementary Figure 9) were performed with the same computational parameters described above but using the more computationally tractable PBE functional. Although this functional does not include van der Waals interactions, it reproduces adsorption energy differences between different adsorbed states. We therefore expect inclusion of vdW interactions would stabilize each adsorption mode similarly, preserving the much stronger binding of the pyr-H^+^ in the sigma-mode bonding and rather weak binding of non-hydrogenated pyr in either sigma or brim bonding modes.

### Data availability

The data that support the findings of this study are available from the corresponding author on reasonable request.

## Electronic supplementary material

Supplementary Information

Peer Review File
