# Supplementary material for: Visualizing hydrogen-induced reshaping and edge activation in MoS2 and Co-promoted MoS2 catalyst clusters
Source: Nat Commun. 2018 Jun 7;9:2211. doi: 10.1038/s41467-018-04615-9 (PMC5992198; doi:10.1038/s41467-018-04615-9)
Supplement: Supplementary file 1 — Supplementary Information [file 41467_2018_4615_MOESM1_ESM.pdf]

PEER REVIEW FILE

Reviewers' comments:

Reviewer #1 (Remarks to the Author):

The article investigates hydrogen-induced particle reshaping in MoS_2 and Co-promoted MoS_2 nanoparticles. The authors first show STM analysis on the edge structures of MoS_2 and CoMoS before and after hydrogenation and demonstrate how those structures change upon hydrogenation within particles of various sizes. An interesting but not quite convincing point is that the authors claim the formation of S-H group on r-CoMoS, but not on r-MoS_2, which also seems important in their attempt to correlate with active site structure in the industrial HDS process. I think, this is overall an intriguing paper but requires major revision before the publication in Nature Communications. My questions are as follows:

1. The structures of s-MoS_2 and s-CoMoS are, with no doubt, clearly presented and carefully analyzed, also in their previous studies. The analysis in Fig. 2c seems difficult to comprehend because the authors did not give detailed structural models for both r-MoS_2 and r-CoMoS. At first sight, it looks contradictory that the hydrogenation reaction produces more S edges. This should be explained in more details along with atomic STM images for r-MoS_2 and r-CoMoS before coming to statistical analysis.
2. The authors show in Fig. 1 that the orientations of s-MoS_2 are dependent on the size and edge structure. But in Fig. 2a, both the largest and the smallest s-MoS_2 particles are pointing to the same direction. Why? Are there any bulk-truncated hexagonal s-MoS_2 particles?
3. In this study, the authors increased the H_2 partial pressure to 10^{-4} mbar, which is two orders of magnitude higher than their previous studies. This brought the question that whether the reduced structure observed is relevant to the structure under industrial conditions and a high pressure of a few torr H_2 will bring new changes after reduction. How stable is CoMoS?
4. The authors have suggested a size effect in s-MoS_2. Are there any size effect in r-MoS_2 and r-CoMoS?
5. The determination of edge structure is not sufficient by line profiles. STM simulation would make the structural determination of r-MoS_2 and r-CoMoS more convincing. Also, the adsorption of a single pyridine molecule cannot justify the presence of S-H group at r-CoMoS. Some vibrational spectroscopy would be necessary to provide the fingerprint of S-H group.

Reviewer #2 (Remarks to the Author):

see attached

Reviewer #3 (Remarks to the Author):

This is a very interesting work which reported the HDS induced restructuring and the related analysis at atomic scale. Authors observed restructuring of edge of MoS2 in HDS and the difference between MoS2 and MoCoS. DFT calculations were used to understand their findings. The following points need to be addressed before considering acceptance.
(1) The title should be specific. STM, the main technique used here should be added to distinguish from TEM observation. Particle is not a specific term. Nanocluster is more appropriate. It should be included in the title. Particles were used twice in the title.
(2) Line 85, “in the range of 10^-4 mbar at 400C”. The pressure is not specific. I believe it is at 10^-4 mbar
(3) Early studies of restructurings such as bimetallic nanoparticles and vicinal surface Pt were not put in context in the discussion of restructuring of edge of MoS2 nanoclusters.
(4) The statement of restructuring of s-MoS2 to r-MoS2 is clearly supported by STM images. However, the lack of restructuring from s-CoMoS to r-CoMoS cannot be clearly seen from Figure 2b.
(5) The number of nanoclusters were used in the statistical account in Figure 2c should be described for each of the four statistical accounts in the caption. What are the confidence level?.
(6) Details and methods of statistical accounts should be added to methods.
(7) STM is the main techniques used in this work. XPS is sensitive for the change of binding from qualitative point and also good for uncovering the formation of sulfur vacancies from quantitative point. Photoemission feature would be important information to support the conclusion.

Reviewer #2 (Remarks to the Author):

The authors study the shape and termination of (Co-promoted) MoS2 nanoparticles supported on gold, using scanning tunneling microscopy and density functional theory.

According to their results, the authors show a selective structural transition of completely sulfided, Au-supported, MoS_2 (s-MoS_2) nanoparticles upon exposure to reductive conditions. This structural transition, from triangular to hexagonal nanoparticles, has been already documented in previous literature by the authors. However, in this case, they also demonstrate that the structural transition seems to be specific to MoS_2 only, while edge activated CoMoS nanoparticles retain a hexagonal symmetry when in a sulfided (s-CoMoS) or reduced state (r-CoMoS).

The authors thoroughly discuss the STM characterization of the r-MoS_2 and r-CoMoS nanoparticle edges. They also give atomic-level details on the presence of sulfur vacancies, through a proper use of STM line scans. For MoS_2, experiments and DFT seems to be generally in good agreement and in agreement with the past literature. The case of CoMoS seems to be more complex and discrepancies between experiments and DFT-calculated phase diagram arise.

In particular, the authors show the presence of "quenched" edges in r-CoMoS (i.e. zones of attenuated intensity in Fig. 3c) that were explained as arising from the S interaction with the gold support. The authors suggest that the presence of adsorbed H at the edge restores the original unquenched state, due to the weakened S-Au interaction. This explanation seems to be sound, as hydrogen is effectively found to be present on the edge through pyridine titration. However, this finding is in contrast with the edge composition predicted by the phase diagram (S50H0 in Fig. 5). According to the latter, the structure should not show any adsorbed hydrogen. The authors try to justify this behavior invoking kinetic effects that prevent hydrogen to desorb as H_2.

It should be noticed that the same S-Au interaction responsible for the quenching effect could be expected also on the unactivated r-MoS_2 (as stated by the authors on line 302); yet in this case no quenched edges were found. This could mean that these differences may be explained in terms of the intrinsically different chemical properties of Co and Mo that here is not analyzed in depth.

The manuscript is well-written and the methodology employed is solid. The topic addresses an important aspect for modelling hydrodesulfurization on Co/MoS2 particles, which is of great interest for the catalysis community, even though real catalysis does not utilize Au as the support. The article should be publishable in NATURE COMMUNICATIONS, provided the authors address the following points:

1. The authors should provide additional information regarding the size of their surface models. The distance between periodically repeated MoS2 nanoparticles is of particular interest to exclude artificial image-image interactions.
2. The second equation in the SI should read "del n = 2n_Mo – n_S". The authors should check, whether this is a typing error or whether it affects the results of their study.
3. To potentially resolve the discrepancy between DFT and experiments about the presence of kinetically trapped hydrogen on the edge, it would be interesting to perform calculations for evaluating the diffusion barrier of hydrogen along the edge. An easy diffusion would substantiate the DFT prediction of the S50H0 structure. High diffusion barriers would instead substantiate the hypothesis of kinetically trapped hydrogen, difficult to desorb as H_2.
4. The STM collected after pyridine titration is discussed by the authors ~ line 267. In particular, they say that: "The outermost edge features in the STM images may reflect a complex

convolution of electronic and geometric structure of the adsorbed molecule, but the symmetry and size agrees well with pyr-H^{+}". In order to have an unambiguous answer on the presence of pyr-H^{+}, it could be interesting to perform explicit DFT calculations on the adsorption of pyridine on the CoMoS hydrogenated edge. In other words, I would confirm the adsorption structure proposed in the empirical sketch of Figure 6b by means DFT. This would allow also to understand if an effective S-Au interaction occurs in presence of pyridine/pyr-H^{+}, confirming the explanation proposed by the authors in lines 269-271.

5. Provide charge density plot to better elucidate the interaction between S and Au, which according to the authors, is responsible for the quenched edge effects.
6. In Figure 1d, the white text is very difficult to read. The authors might want to use a darker outline to increase readability.

Responses to Reviewer Comments:

Reviewer #1:

1. The structures of s-MoS_2 and s-CoMoS are, with no doubt, clearly presented and carefully analyzed, also in their previous studies. The analysis in Fig. 2c seems difficult to comprehend because the authors did not give detailed structural models for both r-MoS2 and r-CoMoS. At first sight, it looks contradictory that the hydrogenation reaction produces more S edges. This should be explained in more details along with atomic STM images for r-MoS2 and r-CoMoS before coming to statistical analysis.

Reply: We refer to question 2 which addresses both question 1 together with question 2.

2. The authors show in Fig. 1 that the orientations of s-MoS2 are dependent on the size and edge structure. But in Fig. 2a, both the largest and the smallest s-MoS2 particles are pointing to the same direction. Why? Are there any bulk-truncated hexagonal s-MoS2 particles?

Reply: Thank you for pointing out this problem, which made us aware that Figure 1 and the method we use to quantify the reshaping this could be misjudged from the illustrations. We believe the origin of the confusion could stem from the original Figure 1, where one could get the impression that particles with the Mo edge termination and S edge termination could be distinguished alone from their orientation of triangular particles pointing up or down. This would be the case when constructing the model from a bulk truncation of an MoS_2 sheet. However, it is not the case for the supported system, as the MoS_2 nanoclusters can grow in two different orientations on the Au support due to fcc and hcp stacking domains formed by the reconstructed Au(111) surface. Hence, the same Mo terminated triangular clusters can be oriented pointing up and down within the same image, without having different edge terminations. This can be seen from the two types of orientation of the s-MoS_2 triangles in figure 2a, where atom-resolved STM images confirm that same edge terminated MoS_2 triangles can be found for both orientations. The edge types are therefore assigned entirely on the basis of atom-resolved STM images (like in Fig 1) for each particle in the statistical plot and the conclusions on their reshaping are reached by careful analysis of the specific amounts of each edge in the particles.

We do not see any significantly truncated s-MoS2 particles, which can be seen from the histogram in Figure 2c. Here s-MoS_2 is generally rather perfect triangular (f~1 (small s-MoS_2) or f~0 (big s-MoS_2) and there are no counts at or around f=0.5.

<u>*Action:*</u> *To avoid the risk of confusion we added the following description to the main text:*
"It is possible to distinguish the Mo-edges and S-edges terminated clusters from atomic resolved STM images (Figure 1-a and 1-b), however the overall orientation of the triangular cluster on the substrate does not directly reflect the nature of the edge termination as the MoS_2 clusters can grow in two different orientations due to the alternating stacking domains of the reconstructed Au substrate."

We also changed Figure 1 so that both the Mo terminated and the S terminated models are pointing in the same direction to make clearer that the relative orientation of the clusters does not implicitly give information on the type of edge termination. We have added a small black triangle in the basal plane of each cluster in Figure 1-a-b-c to emphasize the symmetry of the basal plane lattice. Furthermore we have added an atomic resolved STM image of both r-MoS_2 and r-CoMoS in Figure 2 and added a more detailed discussion in the discussion section concerning Figure 2. Furthermore we color coded the small cartoons in the top of Figure 2c to emphasize that it is the termination of the cluster and not the orientation that we consider, as explained above.

3. In this study, the authors increased the H2 partial pressure to 10-4 mbar, which is two orders of magnitude higher than their previous studies. This brought the question that whether the reduced structure observed is relevant to the structure under industrial conditions and a high pressure of a few torr H2 will bring new changes after reduction. How stable is CoMoS?

Thank you for this relevant question. Overall, the conclusion of our paper is indeed that the absolute pressure strongly defines which edge structures are exposed for both MoS_2 and CoMoS. We were quite surprised to see how a pressure increase by only 2-3 orders of magnitude compared to previous findings triggered a significant change to the morphology and edge structure of the nanoclusters. This pressure increase may seem like a small step, but working

with ultra-high-vacuum equipment such as STM, this is beyond usual pressure tolerances. The very relevant question to ask then is what happens when the pressure is increased even further towards working catalytic conditions? All literature in the field agrees that CoMoS and MoS_2 particles themselves are stable and the response should be found at the CoMoS and MoS_2 edges which adopt S and H coverages that are determined by the chemical potential of mainly H_2 and H_2S. For example, extensive in-situ EXAFS and IR-work has been used for characterize CoMoS catalysts under working conditions (see e.g. the recent book by Toulhoat, H.; Raybaud, P., Catalysis by Transition Metal Sulphides. Editions Technip, Paris: 2013). Also recently, high-resolution STEM-EELS spectroscopy (Y. Zhu, Angew. Chem. Int. Ed. 2014, 53, 10723 –10727) was used to verify the position of Co in CoMoS particles after being exposed to industrial conditions.

A main conclusion in our work is that the reduced r-MoS_2 and r-CoMoS edge structures formed in our experiment are stable over a wide range of conditions, even up to pressures relevant for HDS catalysts. In fact, r-MoS_2 and r-CoMoS cluster are formed at marginally more reducing conditions than HDS. Our DFT calculations accurately predict the transition we see experimentally, i.e. that the chemical potential of hydrogen in the experiment stabilizes reduced S coverages. When extrapolated to HDS conditions, the thermodynamic prediction is that no further S reductions happens, but instead H adsorption may become possible. The major difference between experimental model conditions and HDS conditions, is therefore mainly concerned with an accurate description of H species. New advanced techniques such as ambient-pressure STM and XPS (see ACS Nano 2015, 9, 9322-9330) might be useful to shed light on this issue and our present methods and findings will be instrumental for such clarifications.

4. The authors have suggested a size effect in s-MoS2. Are there any size effect in r-MoS2 and r-CoMoS?

Reply: *Yes, r-MoS_2 implicitly contains the same size effect as for s-MoS_2, since all clusters smaller than a size representing 6 Mo atoms along the cluster edge are S-edge terminated irrespective of the experimental conditions. Since the main structural effect of reducing conditions is to stabilize S edges relative to Mo edge, already fully S edge terminated particles stay this way after H_2 exposure. The data is presented in each the two upper panels of Figure 2c,*

in which small r-MoS_2 particles (n < 6) stay terminated exclusively in S-edges (f~1), whereas bigger r-MoS_2 (n≥6) are terminated by truncated shapes. (f~0.3).
We have analyzed our CoMoS data in depth and no such size effect is observed for neither s-CoMoS nor r-CoMoS. This we attribute to the fact that Co substitution itself leads to a very strong stabilization of the S edges, which dominates the edge stability effects for different cluster sizes.

Action: *We have added this important point to the discussion of the large and small r-MoS_2 nanoclusters: "Small r-MoS_2 nanoclusters (n ≤ 6) hence remain a perfectly S-edge terminated structure (f ≈ 1) whereas large r-MoS_2 nanoclusters (n > 6) is terminated in a combination of Mo- and S-edge (f≈0.3). No similar size effect is observed for neither s- nor r-CoMoS nanoclusters as the Co substitution appears to be the dominating factor of the relative edge stability of CoMoS."*

5. The determination of edge structure is not sufficient by line profiles. STM simulation would make the structural determination of r-MoS2 and r-CoMoS more convincing. Also, the adsorption of a single pyridine molecule cannot justify the presence of S-H group at r-CoMoS. Some vibrational spectroscopy would be necessary to provide the fingerprint of S-H group.

Reply: *The suggestion to include simulated STM images to further determine the structure of r-MoS_2 and r-CoMoS is a valid point. STM simulations can indeed contribute to the assignment of edge types, and we already make use of this. STM simulation were previously successfully used in this respect for edges of s-MoS_2 (see references 7,20,26-28). More recently for r-MoS_2 clusters by Bruix et al. Faraday Discuss. 188: 323-343 (2016), Bruix et al., ACS Nano, 9, 9, 9322–9330 (2015) has included simulated STM data for the relevant S and H coverages of the Mo edge including the ones claimed in our study. These simulations support our interpretation of atom-resolved STM images of the MoS_2 edges. STM simulations of the Co S edge for various S coverages are also found in reference 7. However, we acknowledge that simulated STM images are missing for the important configurations where the r-CoMoS S edge structure it interacting with H. Therefore, we have performed these simulations which is now in the Supplementary Figure S8.*

We agree with the reviewer that vibrational spectroscopy would be a possible way to determine the presence of the S-H groups on CoMoS. IR spectroscopy was previously extensively used for this purpose with great success on industrial style high-surface area samples (e.g. Rangarajan et al., ACS Catal. 2016, 6, 2904−2917). In fact, previous IR spectroscopy work on pyridine adsorption of S-H sites leading to pyridinium was the direct inspiration for our experiment using pyridine as a molecular marker to pinpoint the Brøndsted acid S-H sites in our STM studies. However, the concentration of S-H groups in this sample is likely so low that typical IR spectroscopy approaches would fail to capture a convincing signal. Instead, we exploit the strength of the STM as a local probe technique by adsorbing pyridine as a selective molecular marker to directly probe the position of the S-H groups. In addition, we must also emphasize that the process is quite well-understood theoretically as pyridine adsorption is calculated to be stable on the Co S edge upon the formation of the pyridinium ion on a S-H group (see also our newly added discussion and calculations described in our reply to reviewer 2 question 4). From these combined results we find that pyridine adsorption is a very strong indicator of the presence of individual S-H groups on the Co S edge.

A specialized, but potentially very powerful approach to directly detect S-H stretching energies on the edge sites would be through inelastic electron tunneling spectroscopy (IETS). However, examples of this highly specialized approach on complex samples such as supported nanoparticles are extremely scarce and the experiment requires dedicated cryogenic STM instrumentation that is not currently available to us.

Action: *We have now included STM simulations of the r-CoMoS 50%S Co S edge into our Supplementary Material (Figure S8). The additional simulations qualitatively confirm that the Co S edge starts with a reduced brim intensity in the case of 50%S 0%H Co S edge whereas the H adsorbed on the S edges restores the brim and lifts up the outer protrusions. We refer to these images in the main article. It is worth noting, that the Tersoff-Hamann approach to simulate STM images from the DFT calculated local density of states has well-known limitations for semiconductor materials such as MoS_2, that complicates a further direct quantitative comparison of line scans heights with the experimental ones in figure 3.*

Reviewer #2:

1. The authors should provide additional information regarding the size of their surface models. The distance between periodically repeated MoS2 nanoparticles is of particular interest to exclude artificial image-image interactions.

We agree that this is important information and we have provided a more detailed description in the method section.

2. The second equation in the SI should read "del n = 2n_Mo – n_S". The authors should check, whether this is a typing error or whether it affects the results of their study.

Thank you for pointing this out, the typo is now corrected.

3. To potentially resolve the discrepancy between DFT and experiments about the presence of kinetically trapped hydrogen on the edge, it would be interesting to perform calculations for evaluating the diffusion barrier of hydrogen along the edge. An easy diffusion would substantiate the DFT prediction of the S50H0 structure. High diffusion barriers would instead substantiate the hypothesis of kinetically trapped hydrogen, difficult to desorb as H2.

Reply: *Thank you for this very useful feedback. We agree that quantitative arguments for the proposed trapped hydrogen on the edges are needed. Hydrogen dissociation is a key step in the modelling of HDS processes and a very recent publication by a collaborating group (M. Šaric´ et al., Journal of Catalysis 358 (2018) 131–140) calculates the specific energy profile of dissociating H_2 on a 50% S covered Co S edge to form 2 S-H groups on the edge (see figure below). The activation energy for the reverse process in which the two adsorbed hydrogen atoms recombine on the edge to form H_2 can be found in the same graph to be about 1.1 eV. With this barrier and assuming a pre-exponential factor of maximum 10^{13}, kinetic trapping of H on the Co S edge would be effective at room temperature as stated in the experiment. We believe this value of the desorption barrier is quite consistent with our observation of a moderate amount of S-H groups on the Co S edges.*

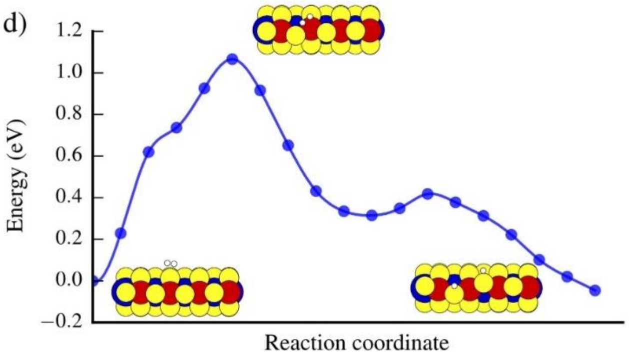

Figure 1: Figure 10d of the article by M. Šaric′ et al. Journal of Catalysis 358 (2018) 131–140 showing the calculated energy profile of dissociating H_2 on a Co S edge

<u>Action:</u> *We have now added the following discussion and references in the claim of trapped H on the Co S edge: "In agreement, H_2 recombination from S-H groups on the Co S edges has an activation barrier of approximately 1.1 eV (M. Šaric′ et al., Journal of Catalysis 358 (2018) 131–140 +*
M. Sun et al., Journal of Catalysis 233 (2005) 411–421) which strongly supports the presence of kinetically trapped hydrogen on the Co S-edge"

4. The STM collected after pyridine titration is discussed by the authors ~ line 267. In particular,they say that: "The outermost edge features in the STM images may reflect a complex convolution of electronic and geometric structure of the adsorbed molecule, but the symmetry and size agrees well with pyr-H+". In order to have an unambiguous answer on the presence of pyr-H+, it could be interesting to perform explicit DFT calculations on the adsorption of pyridine on the CoMoS hydrogenated edge. In other words, I would confirm the adsorption structure proposed in the empirical sketch of Figure 6b by means DFT. This would allow also to understand if an effective S-Au interaction occurs in presence of pyridine/pyr-H+, confirming the explanation proposed by the authors in lines 269-271.

<u>Reply:</u> *Thank you for this suggestion. In addition to experimental IR-spectroscopy studies, there are in fact already extensive modelling studies that support our claim that pyr-H^+ is formed upon pyridine adsorption on a hydrogenated edge. Such calculations have been reported in a study Rangarajan et al. (ACS Catal. 2016, 6, 2904−2917) in which the author determines the energies*

and activation barriers for forming the pyridinium ion upon pyridine adsorption on a hydrogenated 50%S Co S edge (Figure 2 below). Here they first of all find that the formation for the adsorbed pyridinium ion is thermodynamically favorable and that the pyridinium formation has no energy barrier. Hence these calculation clearly concludes that pyridine adsorption on the hydrogenated edges indeed form the pyridinium ion as proposed in our empirically based ball model in the original manuscript.

Initial state	Transition state	Final state	Activation barrier
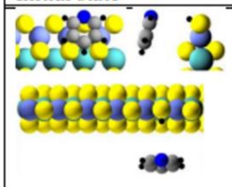 ΔE = 0.0 eV	No Transition state	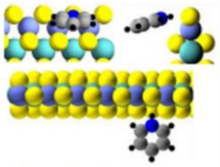 ΔE = -0.62 eV	0.00 eV

Figure 2: Taken from the supporting information of the article by Rangarajan et al. The calculations showing the adsorption energy for adsorbing the pyridine on a protonated Co S edge resulting in the formation of an edge-bound pyridinium ion.

Rangarajan et al. do not specifically include the Au support. However the Au substrate is not expected to influence the adsorption and formation of the pyridinium ion as the main contributors to this adsorption are the Brøndsted basicity of gas phase pyridine, which is not affected by Au, and the strength of the S-H bond. The latter will most likely be very similar for extended CoMoS edges and the supported NP because the chemical potential of H at which the hydrogenated phase appears is very similar for both cases (compare Figure 3 below from Moses et al. for an extended freestanding CoMoS edge to our phase diagram (Figure 5a)).

P.G. Moses et al. / Journal of Catalysis 268 (2009) 201–208

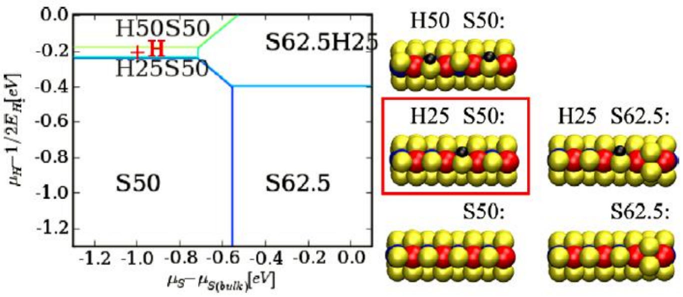

***Figure 3**: Phase diagram of* the extended freestanding CoMoS edge

To fully demonstrate that the Au support does not change the nature of pyridine/pyridinium adsorption we have taken your suggestion and calculated the adsorption energies of pyridine on

hydrogenated but also not-hydrogenated Co S-edges (Figure 6b + S9 of the revised manuscript). Our findings agree with the expected and are 1: adsorption of non-protonated pyridine is not stable 2): protonation stabilizes the adsorption making the adsorption favorable and 3): the most stable configuration of pyr-H^+ is in an upright on-edge adsorption. The calculations suggest only the re-initiation of the Au-S bond in the case of the brim-adsorption, however the neighboring S-atoms in the upright, on-edge adsorption (Figure 6b in the revised manuscript) still remains shifted downwards which may explain the reported observation referred to in the original manuscript (lines 269-271). However, after having discarded the original empiric ball model we find that this speculation is less relevant in the discussion and it has hence been left out in the revised version. We now refer to these new calculations when discussing the pyridine adsorption rather than the empiric ball model (which has been discarded), and we included all additional calculated configurations in the Supplementary Figure S9.

<u>Action:</u>
To further justify the formation of the pyr-H^+ on the hydrogenated Co S edge as originally suggested in the empiric ball model we now replaced the empiric ball model with the calculated stable adsorption configuration (Figure 6b) and added the following text and references:

*"We attribute the feature adsorbed on the edge to the facile adsorption of pyridine on S-H groups on the Co S-edge resulting in the formation of bound pyr-H^+. This is consistent with DFT studies showing that the formation of pyr-H^+ on the hydrogenated 50%S Co S-edge happens with zero activation energy and is thermodynamically favorable (Reference: 25: Rangarajan, S. et al. ACS Catal. 2016**,** 6 (5), 2904-2917). Our DFT calculations further show that the pyridine adsorption of the Co S-edge of our Au supported system is only favorable in the case of the formation of pyr-H^+ ion (Figure 6-b), whereas pyridine adsorption alone on both protonated and non-protonated Co S-edges is significantly less favorable (Supplementary Figure S9)."*

We changed figure 6b from the empiric ball model, and replaced it with the calculated on-edge adsorption configuration of pyr-H^+ which is also the most stable configuration found. The remaining calculated configurations are included in the Supplementary Figure S9.

5. Provide charge density plot to better elucidate the interaction between S and Au, which according to the authors, is responsible for the quenched edge effects.

Reply: Reviewer 1 made a similar point in his/her question 5 which further emphasizes the need of such simulated STM data (charge density plot), and we refer to the replies and actions taken in our reply number 5 to reviewer 1.

6. In Figure 1d, the white text is very difficult to read. The authors might want to use a darker outline to increase readability.

Reply: Thank you for pointing this out. We have changed the figure so the text is now outside the basal plane of MoS_2.

Reviewer #3:

(1) The title should be specific. STM, the main technique used here should be added to distinguish from TEM observation. Particle is not a specific term. Nanocluster is more appropriate. It should be included in the title. Particles were used twice in the title.

Reply: We have now changed the title to "Visualizing hydrogen-induced reshaping and edge activation in MoS_2 and Co-promoted MoS_2 catalyst clusters using Scanning Tunneling Microscopy" and changed the word "particle" to "cluster" throughout the paper.

(2) Line 85, "in the range of 10^-4 mbar at 400C". The pressure is not specific. I believe it is at 10^-4 mbar

Reply: We agree and we have changed the text as suggested.

(3) Early studies of restructurings such as bimetallic nanoparticles and vicinal surface Pt were not put in context in the discussion of restructuring of edge of MoS2 nanoclusters.

<u>Reply:</u> Thank you for pointing this out, we have now added citations to some of the early work in the field of gas induced reconstructions, noting that the effect for compound nanoclusters such as sulfides are less well understood than corresponding metallic systems.

(4) The statement of restructuring of s-MoS2 to r-MoS2 is clearly supported by STM images. However, the lack of restructuring from s-CoMoS to r-CoMoS cannot be clearly seen from Figure 2b.

<u>Reply:</u> We agree with the reviewer that direct inspection of the images does not immediately lead to a definite conclusion that no reshaping takes place for CoMoS. This is why the detailed statistical account on many images in Fig 2c is important, as it allows us to quantitatively conclude that shapes for r-CoMoS are unchanged on the basis of a significant statistical material. In response to the points made we have realized that the images could be more representative. We also became aware how the original text suggests that the conclusion on the lack of shape change was based simply on comparing the two STM images of Figure 2-b. This is not the case – the conclusion was made based on the statistics in Figure 2-c, lower panel, which we have now made clear in the text.

<u>Action:</u> We have changed the STM image of r-CoMoS in Figure 2b to an image that more clearly reflects the observed lack of restructuring (See also reply and action taken to reviewer 1, question 1 and 2). In addition, we have rewritten the entire paragraph about the CoMoS reshaping to make clear that the conclusion was built on the statistical analysis and not by just comparing the STM images of Figure 2-b. The part of the text that refers to this question is:

"The ability to distinguish S- and Mo-edges in both s- and r-CoMoS allows us once again to make a statistical analysis of the cluster shape, which is necessary to conclude whether or not an overall shape change occurs. For a large number of individual CoMoS clusters from various areas of the

sample, the length of each edge was measured and the clusters' shape factor ($f = l_S/l_{total}$) was calculated. The statistical distribution of the shape factor, f, (plotted in Figure 2-c, lower panel) surprisingly suggests no statistical significant shape change between the s-CoMoS (dark red bars) and r-CoMoS (bright red bars), which both appear to reflect almost perfect hexagons ($f \approx 0.5$). The hexagonal shape of the CoMoS nanocluster is therefore concluded to be the stable equilibrium shape in both cases."

(5) The number of nanoclusters were used in the statistical account in Figure 2c should be described for each of the four statistical accounts in the caption. What are the confidence level?

<u>Reply</u>: *We agree. We have added the number of nanoclusters for each analysis to the Figure caption as requested. To avoid a too long and complicated caption we have added all additional statistical information requested in this and the following question in the methods section and placed a reference in the corresponding Figure caption***.**

(6) Details and methods of statistical accounts should be added to methods.

<u>Reply:</u> *We agree. A detailed description has now been added to the methods section along with the parameters requested in the previous question*.

(7) STM is the main techniques used in this work. XPS is sensitive for the change of binding from qualitative point and also good for uncovering the formation of sulfur vacancies from quantitative point. Photoemission feature would be important information to support the conclusion.

<u>Reply:</u> *XPS is definitely a powerful technique for probing chemical changes within a sample and can provide information about the sulfur coverage on the edges. Near Ambient Pressure (NAP)-XPS of MoS_2 nanoclusters Au(111) in hydrogen has already been carried out with success by some of us and reported in the work by Bruix et al. in Ref. 13. That study supports the gradual removal of S content on the Mo edges under elevated H_2 pressures. However, the present studies*

also shed new light on these findings, as the emergence of S edges with a distinct chemical shift was not accounted for in these studies. We are in process of developing new dedicated sulfur-tolerant equipment for further XPS studies to address the response of CoMoS clusters, as we find it difficult to obtain beam-time for this important experiment on user facilities due to the contamination issues often seen when using sulfur-containing compounds.

<u>Action:</u> *We have mentioned XPS in our conclusion as important tool to further clarify the response of CoMoS to reaction conditions:*
"In future studies, changes of the CoMoS morphology under H_2 *exposure monitored by spectroscopy techniques such as ambient pressure X-ray photoemission spectroscopy could provide further insight regarding the morphology change dynamics and stable configurations."*

<u>Reviewers' Comments</u>:

Reviewer #1 (Remarks to the Author):

The authors made major revisions and improved the manuscript considerably. I think that the manuscript is now suitable for publication,

Reviewer #2 (Remarks to the Author):

The authors have addressed my questions/concerns appropriately, added relevant references, and therefore the revised manuscript is recommended for publication as is.

Reviewer #3 (Remarks to the Author):

Authors have addressed my concerns and made corresponding changes. I would recommend acceptance to the revised manuscript.
