# Supplementary material for: Visualizing hydrogen-induced reshaping and edge activation in MoS2 and Co-promoted MoS2 catalyst clusters
Source: Nat Commun. 2018 Jun 7;9:2211. doi: 10.1038/s41467-018-04615-9 (PMC5992198; doi:10.1038/s41467-018-04615-9)
Supplement: Supplementary file 2 — Peer Review File [file 41467_2018_4615_MOESM2_ESM.pdf]

Supplementary Material

Visualizing hydrogen-induced reshaping and edge activation in MoS_2 and Co-promoted MoS_2 catalyst clusters

Signe S. Grønborg, Norberto Salazar, Albert Bruix, Jonathan Rodríguez-Fernández, Sean D. Thomsen, Bjørk Hammer, Jeppe V. Lauritsen

Interdisciplinary Nanoscience Center (iNANO), Aarhus University, Gustav Wieds Vej 14, 8000 Aarhus

and

Department of Physics and Astronomy, Aarhus University, Ny Munkegade 120, 8000 Aarhus, Denmark

Corresponding author: jvang@inano.au.dk

Supplementary Figures:

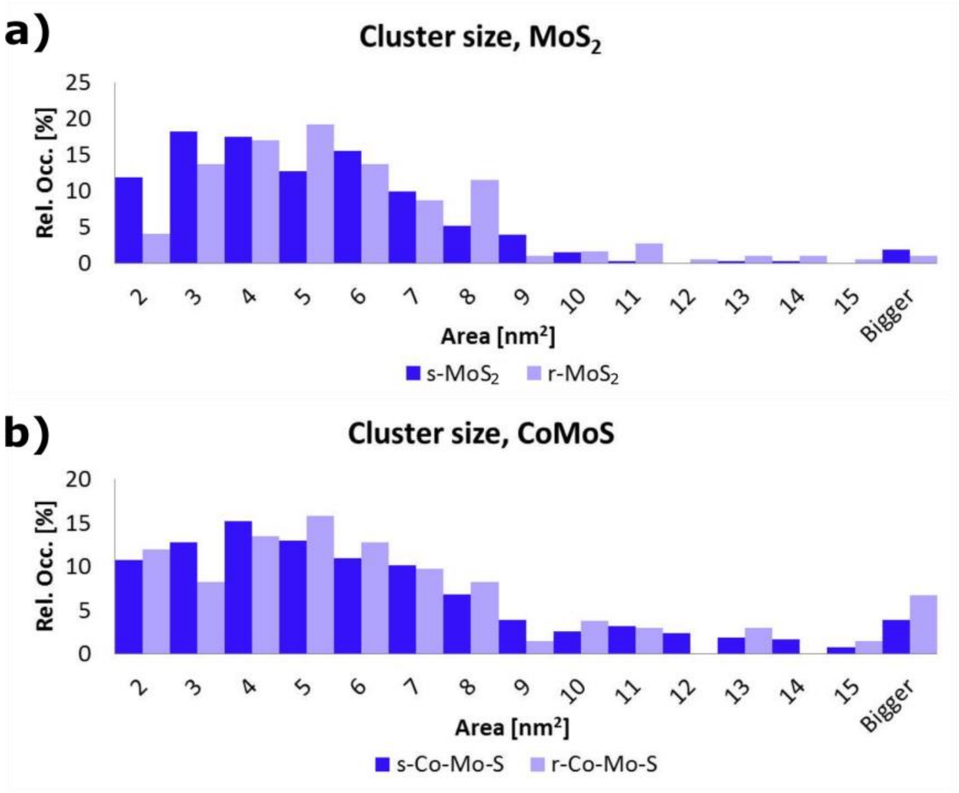

Supplementary Figure 1: (a) Histogram of the size distribution of the sulfided and reduced nanoclusters. (a): MoS_2 and (b): CoMoS.

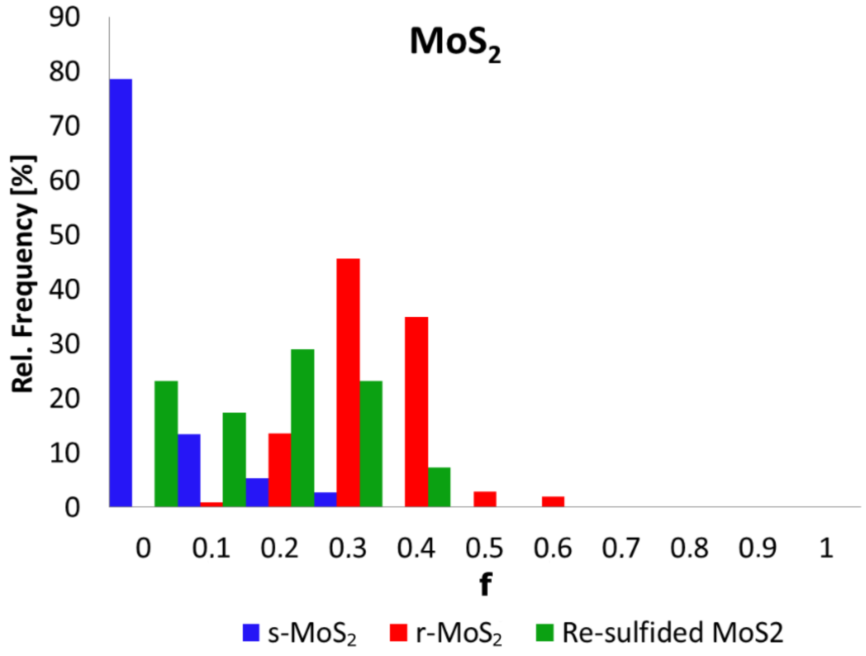

Supplementary Figure 2: Resulfidation effect on shape factor. Plot of the shape factor '*f*' for the cycle of the MoS_2 nanoclusters after sulfidation (*s*-MoS_2), reduction (*r*-MoS_2) and resulfidation in 10^{-6} mbar of H_2S at 673K corresponding to the initial sulfidation.

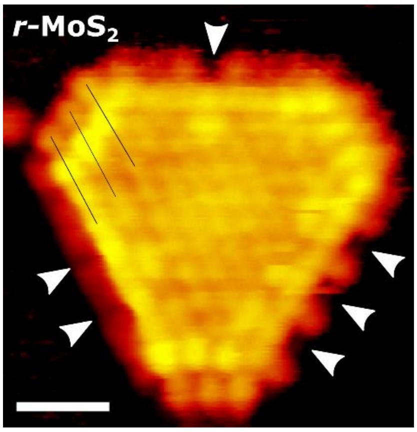

Supplementary Figure 3: *r*-MoS2 nanocluster. Example of atom-resolved STM image (I_t=0.45, V_t=-0.24V) of a *r*-MoS_2 nanocluster with Mo edges that display contrast that can be assigned to edges with less than 50%S coverage, but also parts with a higher than 50% S coverage. The white arrows indicate the position of sulfur vacancies. The black lines superimposed on the cluster illustrates that the protrusions on the shorter S-edges are imaged in-registry with the basal plane S atoms. Scale bar is 1 nm.

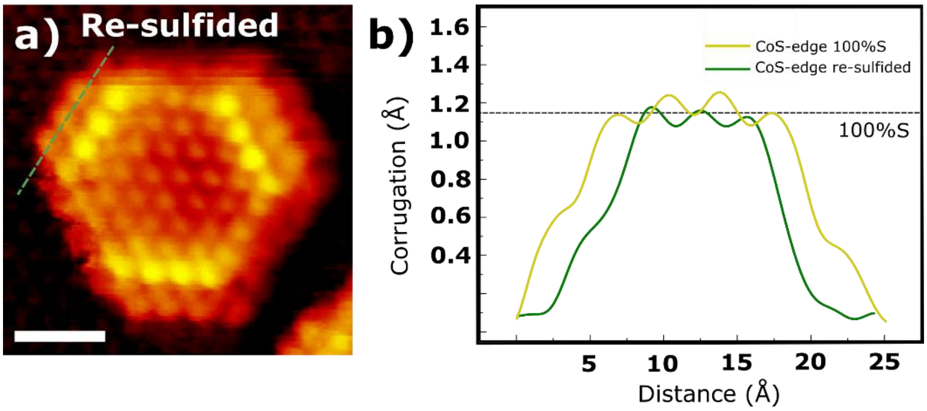

Supplementary Figure 4: Re-sulfided *r*-CoMoS nanocluster. (**a**) Atom-resolved STM image (I_t=0.63nA, V_t=-0.01V) of resulfided *r*-CoMoS showing that resulfidation recovers the original S coverage of the *s*-CoMoS cluster. Scale bare is 1 nm **(b)** Line scans of the resulfided Co S edge comparing *s*-CoMoS (image not shown) with the resulfided edge indicated by the green line in a. Note the edges had a different length.

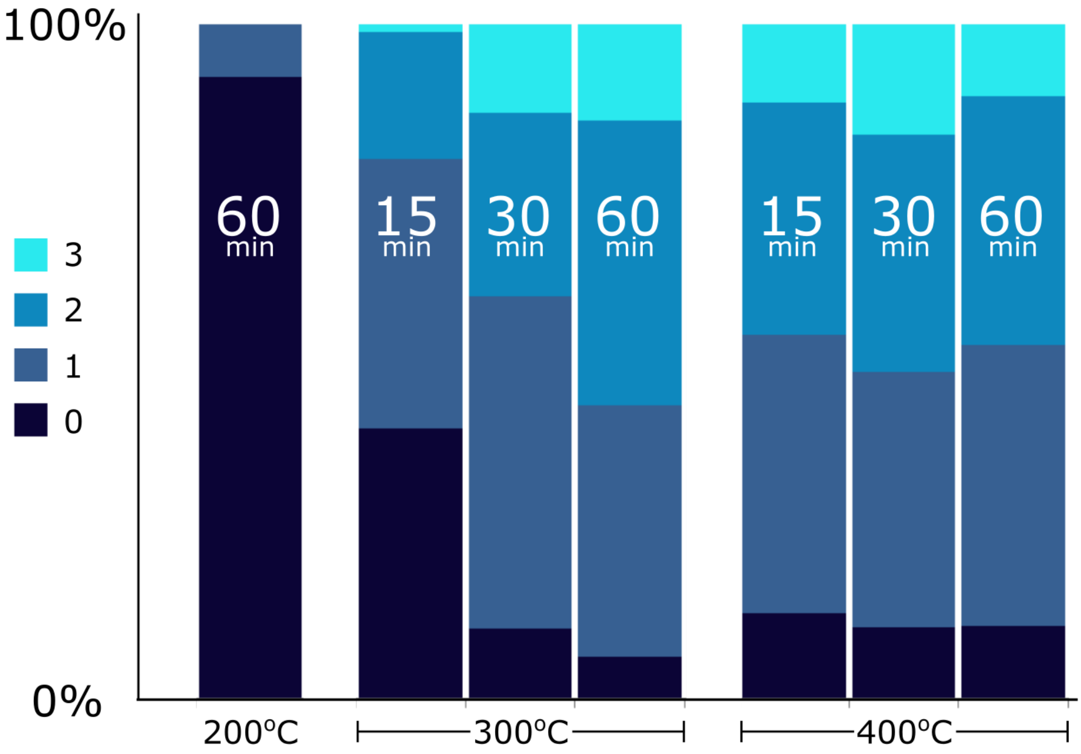

Supplementary Figure 5: Number of quenched edges per r-CoMoS nanocluster**.** Statistical distribution of the number of observed quenched brims per *r*-CoMoS nanocluster for varying H_2 annealing time and temperature as stated in the figure. Non-quenched Co S edges reflect 50% S with H adsorbed, while the quenched Co S edges are 50% S with no H. As a CoMoS hexagon contains 3 S-edges. Partial H coverages (partially quenched edges) were rarely observed, so in the analysis each CoMoS hexagon may generally contain 0, 1, 2 or 3 fully quenched S-edges, where 3 corresponds to no H on any S edges. The numbers reflecting the coloring from dark to light blue, scale as follows: 0 = no quenched Co S-edges to 3 = all Co S-edges quenched.

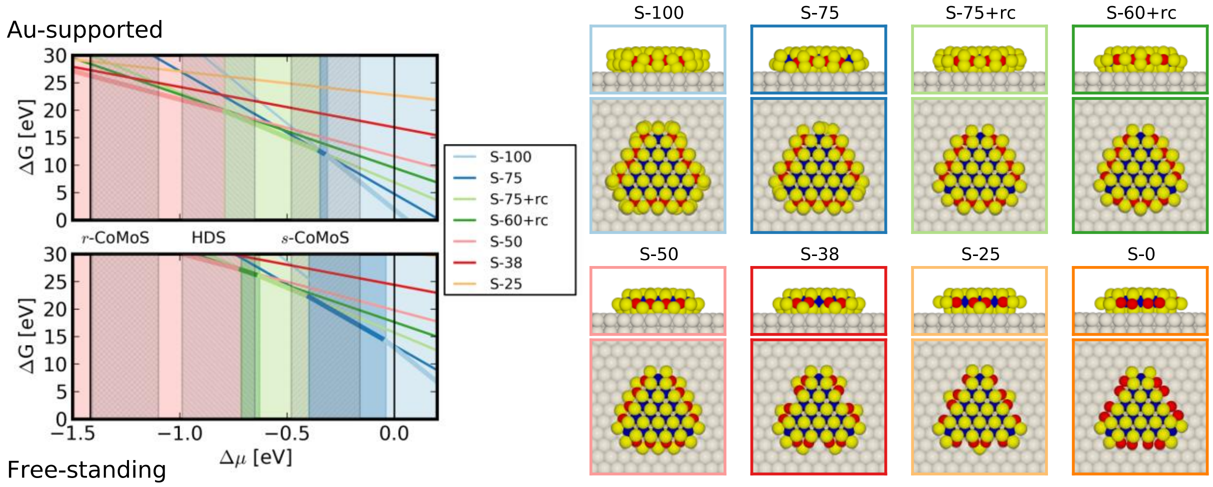

Supplementary Figure 6: Sulfur coverage for CoMoS. **Left:** Phase diagram for the CoMoS nanoclusters for both Au(111)-supported (upper) and free-standing (lower) cases. CoMoS structures are shown (Red: Co, Yellow: S, Blue: Mo, Gray: Au). The number of calculated configurations for each phase diagram is larger than in the main text, where a simpler version with the four most relevant states is presented. The term “rc” in the phase diagram labels refers to states in which the corners (Mo edges) are reduced to 50%. Hashed areas indicate regions of μ_S estimated to be representative of the indicated experimental conditions (i.e. reducing, sulfiding, or HDS) **Right:** Resulting CoMoS structures for the structures in the Au supported phase plot with edge S coverage on the Co S edge ranging from 100%S to 0%.

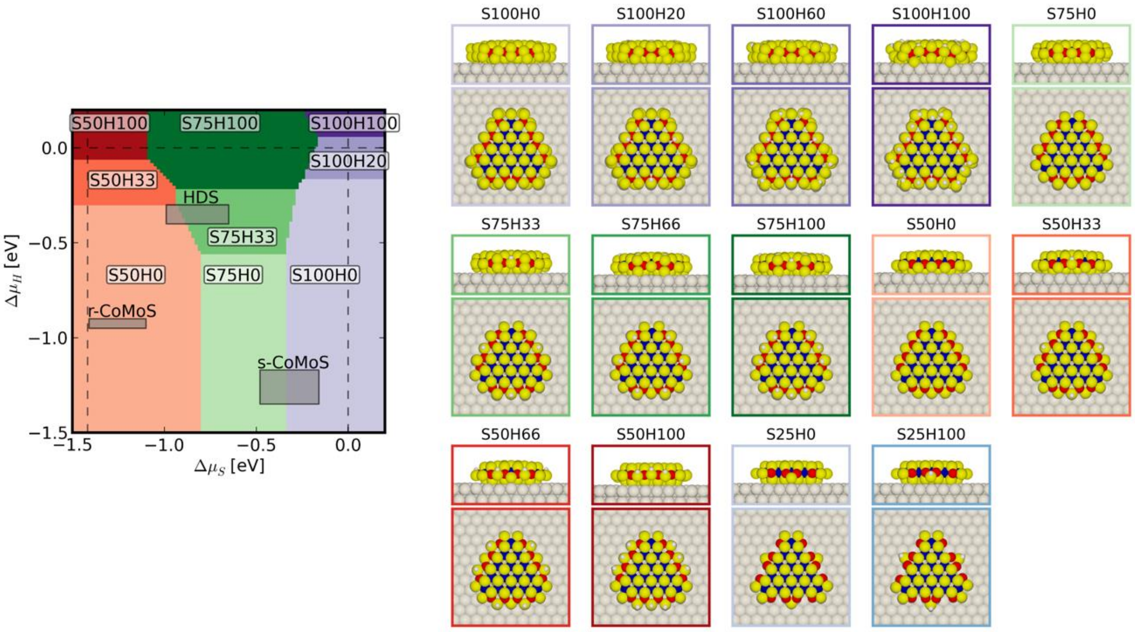

Supplementary Figure 7: Extended phase diagram for the CoMoS S-edge. The shown ball models are all the Au-supported CoMoS nanoclusters used for constructing the phase diagram.

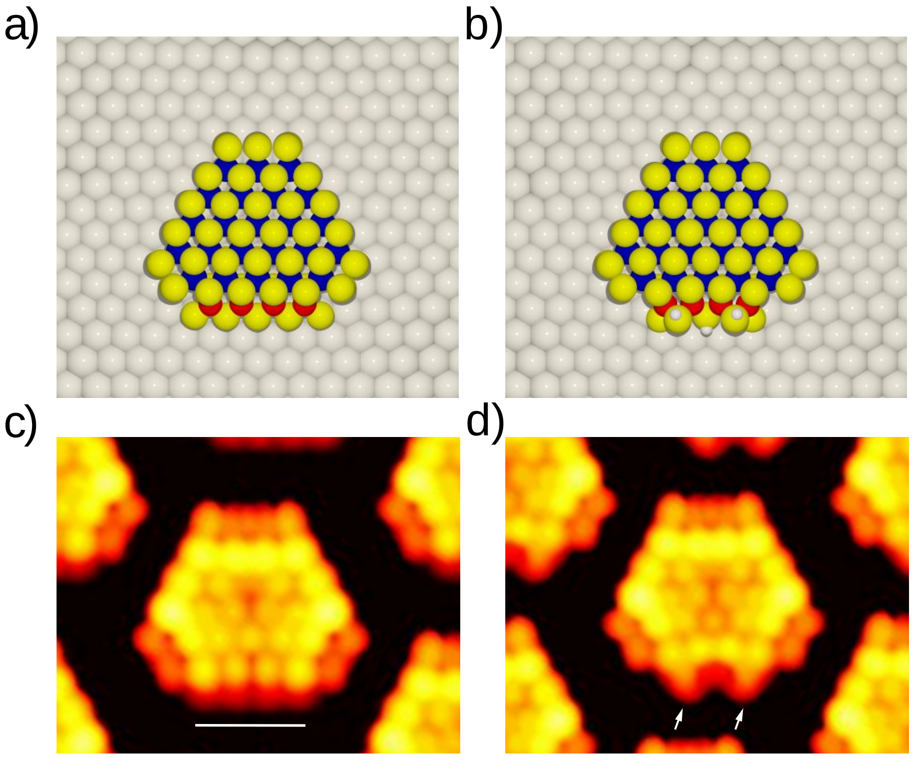

Supplementary Figure 8: Structural models and resulting STM simulations. STM simulations (Teroff Hamann approximation, I_t = 1.7 mA, V_t = -0.80 V) of a CoMoS nanoclusters on Au with 50% S Co S edge with and without hydrogen. To simplify the calculations only one S edge contains Co. a+c) Simulated STM image and corresponding ball model for the 50% S Co S edge without H. The simulated STM image qualitatively captures the lack of bright brim along the edge (Co S edge of model highlighted by the white line parallel to the edge) as observed in the experiment. b+d) simulated STM image and corresponding ball model for the 50% S Co S edge with 3 adsorbed hydrogen atoms. Both a slightly brighter brim and outer S-row protrusions for the hydrogenated state are captured by the model (indicated by white arrows).

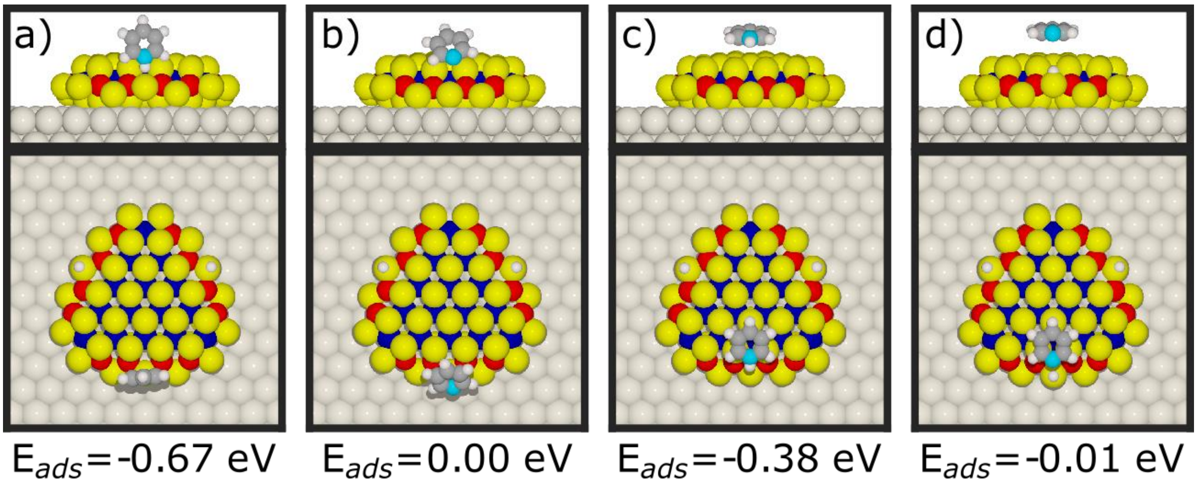

Supplementary Figure 9: Calculated adsorption energies for pyridine adsorption. Various pyridine configurations on the protonated (a, c, d) and non-protonated (b) Co S-edges. a) on-edge adsorption of pyridine on a protonated Co S-edge, leading to the formation of strongly bound pyr-H^+. b) on-edge adsorption of pyridine on a non-protonated Co S-edge c): on-brim adsorption on the Co S-edge leading to pyr-H^+ formation and d): on-brim pyridine adsorption on hydrogenated Co S-edge leading to non-protonated pyr.
